# THE EFFECT OF VISUAL DEPRIVATION DURING COGNITIVE MOTOR DUAL TASK TRAINING ON COGNITIVE FUNCTION IN TYPE 2 DIABETES MELLITUS

**DOI:** 10.12688/f1000research.162466.1

**Published:** 2025-06-17

**Authors:** J Anandh Raj, Ramesh Chandra Patra, Kavitha S, V Subramanyam, K Himabindu, Kilani Kusuma, M.L. Ramya Krishna

**Affiliations:** 1 Lovely Faculty of Applied Medical Sciences, Lovely Professional University, Phagwara, Punjab, 144411, India; 2 Lovely Faculty of Applied Medical Sciences, Lovely Professional University, Phagwara, Punjab, 144411, India; 3 School of Health Sciences, The Apollo University, Chittoor, Andhra Pradesh, 517127, India; 4 School of Health Sciences, The Apollo University, Chittoor, Andhra Pradesh, 517127, India; 5 School of Health Sciences, The Apollo University, Chittoor, Andhra Pradesh, 517127, India; 6 Faculty of Physiotherapy, Meenakshi Academy of Higher Education and Research, Chennai, Tamil Nadu, 600078, India; 7 School of Health Sciences, The Apollo University, Chittoor, Andhra Pradesh, 517127, India

**Keywords:** Type 2 Diabetes mellitus, cognitive function, cognitive motor dual task training, montreal cognitive assessment (MoCA).

## Abstract

**Background:**

Diabetes Mellitus (DM) is a chronic metabolic disorder caused by hyperglycemia, impaired insulin secretion, and insulin resistance. Type 2 diabetes mellitus (T2DM) is associated with an increased risk for cognitive dysfunction. Cognitive motor dual task blindfold training (CMDBT) forces the brain to process motor tasks in one of the four procedural memory centers: the basal ganglia, cerebellum, supplementary motor area, and premotor cortex. Hence, it helps improve cognition in patients with T2DM.

**Methods:**

A randomized control study was conducted on 62 subjects with type 2 diabetes mellitus. Pre-interventional measures were measured using the MoCA scale to assess cognition. The experimental group [n=31] underwent Cognitive motor dual task blindfold training, along with aerobic training. The control group [n=31] received conventional aerobic and resistance exercises. The subjects in both the groups received the intervention for 12 weeks. Post interventional outcomes were measured using the MoCA scale in subjects with T2DM.

**Results:**

Statistical analysis of the data revealed that there was a significant improvement in cognitive function in experimental group A subjects, with a significant difference observed in group A compared to group B. The P value of MoCA was 0.0001 in experimental group A subjects.

**Conclusion:**

Cognitive motor dual-task training (CMDTT) is more effective in increasing cognition in subjects with T2DM. Statistical analysis showed that group A (CMDTT) showed greater improvement in cognitive function than the control group.

## Introduction

Diabetes mellitus comprises a group of chronic metabolic illnesses distinguished as high blood glucose levels resulting from deficiencies in insulin secretion, insulin action, or both. ^1^ Unlike T1DM, T2DM, an autoimmune illness marked by the death of beta cells that produce insulin, is primarily brought on by reduced insulin action, commonly referred to as “insulin resistance,” along with varied degrees of insulin shortage. ^2^ The World Health Organization (WHO) estimated that 422 million people worldwide had diabetes in 2014. Nearly four million fatalities annually are attributed to high blood sugar. ^3^ By 2045, there will be at least 629 million diabetics worldwide. As per the International Diabetes Federation (IDF), 850 billion US dollars were spent on adult diabetes-related medical treatment worldwide in 2017. In India, the prevalence of T2DM is rapidly increasing, with significant public health implications. ^4, 5^

Type 2 diabetes is not only a metabolic disorder but also a significant risk factor for cognitive decline and dementia. ^6– 8^ The mechanisms underlying this association include hyperphosphorylation of tau protein, which is a key feature in insulin dysregulation and Alzheimer’s disease. ^9– 11^ The chronic development of advanced glycation end products (AGEs) may result from increased blood glucose levels. ^12^ Even though the development of AGEs considers the pathophysiology of dementia and Alzheimer’s disease, people with dementia who also have type 2 diabetes have been shown to exhibit more AGEs and microglial activation than people with dementia alone. ^13^ The moderate to severe vasculopathies seen in dementia and type 2 diabetes are largely caused by endothelial dysfunction. Microalbuminuria, an indicator of endothelial dysfunction, is found in individuals with type 2 diabetes and is associated with accelerated cognitive deterioration. Damage to endothelial cells caused by protein buildup on blood vessel walls increases the creation of reactive oxygen species and decreases the production of vasodilatory factors, which lowers cerebral blood flow and causes neurovascular uncoupling and neuronal damage. ^8, 12^ Verbal fluency, executive function, processing speed, memory, and overall cognitive function all diminish more quickly in people with type 2 diabetes. ^14^

In treating cognitive impairment in T2DM, resistance training is used to improve cognitive function as resistance training protects from degeneration in specific subregions of the hippocampus. ^15– 17^ A 6-month multi-modal exercise program has improved cognitive function during rehabilitation. This multi-model exercise program showed good benefits on cognitive deterioration in individuals with type 2 diabetes. ^15, 16^ Physical - cognitive training is a technique used in adults with T2DM along with cognitive impairment, which gave better results in improving the cognitive function. The intervention included moderate-intensity exercises combined with cognitive training. ^17, 18^

Aerobic exercise training improves insulin sensitivity, glycemic control, and cognitive function. These improvements are frequently attributed to adaptations linked to chronic exercise, such as improvements in cardiorespiratory fitness, adjustments to energy balance, and decreases in total or regional adiposity. ^19^ One type of neurological exercise therapy used to help older persons with type 2 diabetes recover from cognitive impairment is the mind-motor approach. Patients with type 2 diabetes saw an improvement in their cognitive function as a result. ^20^ Dual task training: Although cognitive training and exercise are acknowledged as viable methods for addressing cognition, it is feasible that combining the two simultaneously (dual-task) may have complementary or shared mechanisms that enable larger cognitive benefits than either one alone. ^21– 23^ This synergistic approach aligns with the guided plasticity facilitation framework, which emphasizes that simultaneity of physical and cognitive stimulation maximizes neuroplasticity by leveraging exercise-induced molecular changes (e.g., increased BDNF levels) during cognitive task performance. ^24^

By performing a cognitive task concurrently with motor training, a technique known as MCDTT can more successfully fortify the functional connections between motor cognitive brain regions, promoting cerebral cortex activation and improving walking ability. Improving motor dysfunction makes it easier for the cerebral cortex to become active while helping the brain to remodel. ^25^ The ability to walk is a unique behavior that distinguishes humans from other species. Autonomic conscious modulation of casual posture and stress feedback control and information processing through visual, proprioceptive, vestibular, and additional sensory experiences are all part of the control processes. ^26, 27^

External stimulation triggers endogenous brain regeneration strategies that promote cortical activation through motor training and cognitive behaviors as stroke patients gradually regain their functional abilities. It speeds up information processing by encouraging brain restoration and the expansion of cortical neuronal connections. ^25^ Researchers have also discovered, using radiography, that dual-task training improves the hemodynamics of the dorsolateral prefrontal cortex. Simultaneously, central activation decreases following dual-task training, suggesting a reduced processing load following training. ^28, 29^

Blindfolding is a technique used for visual deprivation to train individuals through intuition and sensory substitution. Blindfold is not the end goal, but it helps to stimulate and improve the brain’s abilities. The blindfold technique can boost our brain and balance the left and right hemispheres of the brain, including activating intention capacity, which helps individuals do things with their closed eyes ^30^ Paul Bach-y-Rita et al. was the first to propose the sensory substitution. A non-invasive method for preventing amputation on one sense is sensory replacement, which involves supplying information through a different route. It re-establishes a sense of surroundings in individuals. ^31^

A complete sensory modality conveys information to the visual perception regions of the brain (occipital lobe and visual cortex) during sensory replacement, enabling an individual to comprehend and identify sight. Brain structures associated with various sensory modalities can receive information from a single modality. Information from the touch receptors is transmitted to the visual cortex via touch-to-visual sensory substitution for processing and perception. Individuals’ eyes were closed in the blindfold activity, and they were asked to use touch to determine the colour of the ball/object. Tactile-visual sensory substitution occurs when individuals describe their perceptual experiences with a specific object. Sensory substitution has not only provided impressive practical results among those with visual impairments but has also developed neural plasticity. ^32^

Cognitive-motor dual-task training is a non-pharmacological approach that combines regular mental and physical activity to maintain or improve cognitive skills. Such skills include attention, cognitive flexibility, problem solving, observation, comprehension, intuition, reasoning, and memory. This technique boosts cognitive abilities by strengthening neural connections in the brain, which improves the brain’s ability to process and understand information. Mental exercise sessions focusing on improving cognitive and intuitive skills are known as cognitive training, such as memory, focus, attention, and observation. This can help maintain or improve certain aspects of cognition. ^33^ In T2DM, Cognitive Motor dual task blindfold training (CMDBT) may be beneficial for cognitive function improvement along with motor improvement that result in exercise capacity enhancement along with decreased risk of other types of diabetes onset-associated complications including dementia, diabetic myopathy, sarcopenia, neuropathy. Anticipated hypothesis of proposed study is that CMDBT can improve cognitive function in T2DM individuals. ^11^

## Method

Based on the literature, cognitive motor dual-task training has been conducted on cognition, but there is no adequate evidence in type 2 diabetes mellitus. This study was approved by the institutional ethics committee on 30-12-2023 at the Apollo Institute of Medical Science and Research, Chittoor, Andhra Pradesh, India. Ethics Committee Number: **PG/35/IEC/AIMSR/2023.** The study ensures that All participants provided written informed consent before data collection. The investigator was collected the data regarding baseline assessment of demographic data of name, age, gender, height, weight, duration of the disease, HbA1C and Mini mental state examination score. After the collection of data of base line assessment then the subjects was allocated with random block envelope method to experimental (CMDBT) group A, and conventional (control) group B.

**Research design:** Experimental design- Single blinded Randomized Controlled Trial

**Study population:** Study includes patients diagnosed with Type 2 diabetes mellitus

**Sampling method:** Random sampling method with 1:1 parallel arm allocation ratio with lottery method

**Study design:** Experimental study – Randomized control trial **(Clinical Trial Registry of India no: CTRI/2024/01/061956)**

**Study setting:** The study was conducted at the physiotherapy OPD in the Apollo District Headquarters Hospital, Murukambattu, Chittoor-517001, Andhra Pradesh, India.

**Sample size:** Total sample size: 62 subjects were randomly allocated to each group of 31.

**Sampling method:** Random sampling method, 1:1 ratio parallel with lottery technique.

**Treatment duration:** One session a day, 3 days/week, for 12 consecutive weeks.

**Inclusion criteria:**

• Subjects with T2DM
• HbA1c value above 6.5mmol/dL
• Subjects with education level >5 years (need to read and write).
• 5-10 years of diabetes duration from diagnosis
• Both male and female genders are included.
• Subjects who signed consent and willing to participate.

**Exclusion criteria:**

• Patients who are not willing and not cooperative. Musculoskeletal anomalies
• Pressure sores and Pressure ulcers
• Any exposure to radiological X rays or therapy since past 6 months.
• Any microvascular circulation defects
• Diabetic neuropathy patients
• Patients with unstable vitals
• Patients with cardiac anomalies
• Malignant tumors

**Outcome measures:** Montreal cognitive assessment (MoCA).

### Procedure

In this study, participants who fulfilled the selection criteria were asked to provide written informed consent. Baseline measurements were obtained after obtaining consent. A total of 62 subjects were allotted randomly to group A (experimental) and group B (control) in 1:1 parallel with the lottery method. The subjects who fulfilled the eligibility criteria underwent pre interventional assessment MoCA.

### Experimental group

Participants in the intervention group received a structured, multicomponent training regimen delivered in 3 sessions per week for 12 weeks, totaling 36 sessions. Each session combined progressive cognitive motor dual task blindfold exercises with conventional physiotherapy, all performed while wearing a blindfold on a treadmill to enhance dual-task demands and minimize visual cues. If any discomfort was experienced, participants were allowed rest breaks during the session. Cognitive training targeted working memory (via digit span and word list recall tasks, with sequence length increasing every four weeks), visuospatial skills (through auditory instruction to interpret and set clock times, progressing from standard to oblique and advanced positions), executive function (using serial arithmetic and verbal sequencing tasks with escalating complexity and time constraints), attention (via digit ordering and auditory detection tasks with progressively greater demands), and language processing (including verbal memory and fluency tasks with increasing delay and time pressure). All cognitive motor dual task blindfold exercise were administered in 10 trials per session, with task difficulty systematically increased every four weeks to ensure adaptive challenge. In parallel, conventional physiotherapy comprised resistance training of moderate intensity (50–69% of one-repetition maximum, using a moderate resistance band) targeting the shoulder, elbow, wrist, hip, knee, and ankle joints with 10 repetitions per exercise, as well as aerobic training of moderate intensity (55 to <70% of maximum heart rate) conducted through cycling and treadmill walking. This integrated, dual-task approach was designed to simultaneously enhance cognitive and physical capacities while maintaining consistent dosage and progressive difficulty throughout the intervention period.

### Control group

Participants in the control group received conventional physiotherapy consisting of moderate-intensity resistance training (50–69% of one-repetition maximum) and moderate-intensity aerobic training. Resistance exercises targeted the shoulder, elbow, wrist, hip, knee, and ankle, using a moderate resistance band for 10 repetitions per exercise. Aerobic training was performed at 55–70% of maximum heart rate (HRmax) through cycling and treadmill walking. All interventions were administered once per day, three days per week over a 12-week period, ensuring a total of 36 sessions for each component.

### Statistical analysis

Statistical analysis was performed using IBM SPSS Statistics 30 version ^34^ under subscription version. with a two-tailed alpha level of 0.05 defining significance. Normality of data distribution was confirmed via Shapiro-Wilk tests ( *W* > 0.90 for all groups). Within-group changes in MoCA scores were analyzed using paired *t*-tests, while between-group differences at post-intervention were assessed via independent *t*-tests. Effect sizes were calculated using Cohen’s *d*, interpreted as small ( *d* = 0.20), medium ( *d* = 0.50), and large ( *d* ≥ 0.80). Homogeneity of variance was verified with Levene’s test ( *p* > 0.10 for all comparisons), supporting the use of equal variances assumed in *t*-tests. Clinical significance was evaluated against the established minimal clinically important difference (MCID) of 2.3 points for MoCA in diabetic populations. All data are reported as mean ± standard deviation (SD), with 95% confidence intervals (CI) calculated for mean differences. No adjustments for multiple comparisons were applied, as the study prioritized identifying preliminary effects for future confirmatory trials.

## Results

**Figure 1.**
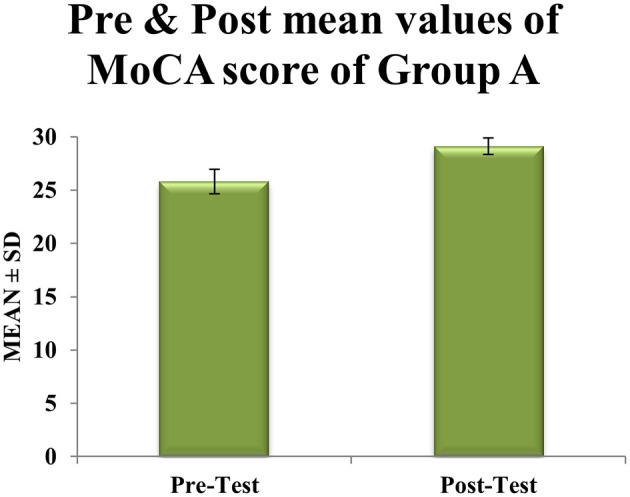
Graphical representation of Means of pre and post values of MoCA within experimental Group A.

**Figure 2.**
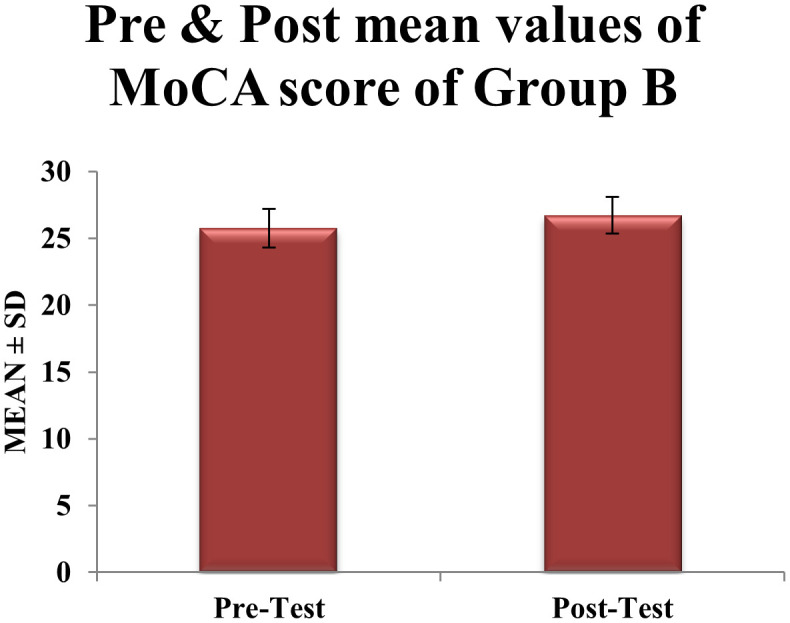
Graphical representation of Means of pre and post values of MoCA within CONTROL Group B.

In CMDBT group (Group-1), 21 (67.7%) males & 10 (32.3%) females had mean, standard deviation of age 51.35±5.43yrs, duration of diabetes had been 9.06 ± 3.57, educational level was 11.81 ± 3.08, HbA1c value was 7.70 ± 1.21, BMI was 27.52 ± 2.22 kg/m ^2^, Mini mental state score was 27.42 ± 1.31. In the Control group (Group-2), 18 (58.1%) males & 13 (41.9 %) females having mean as well as standard deviation of age had been 50.90±5.07yrs, duration of diabetes had been 9.71 ± 3.53, educational level was 11.06 ± 3.26, HbA1c value was 7.81 ± 1.27, BMI was 27.53 ± 1.97 kg/m ^2^, Mini mental state score was 27.35 ± 1.11 participated. Every demographic measure in 2 groups had not been statistically significant ( *p* > 0.05) from one another, demonstrating homogeneity of 2 groups. ^37^ The study revealed significant within-group improvements in cognitive function across both intervention arms. Participants in Group A (Cognitive-Motor Dual-Task Training with Blindfold) exhibited a clinically meaningful enhancement in global cognition, as evidenced by Montreal Cognitive Assessment (MoCA) scores increasing from a pre-intervention mean of 25.81±1.74 to 29.13±0.76 post-intervention ( t=6.32 , df=30 , p<0.0001 ) ( Table 1). This 3.32-point gain exceeded the minimal clinically important difference (MCID) of 2.3 points for MoCA, accompanied by a 56% reduction in score variability (standard deviation: 1.74 to 0.76), indicating consistent treatment effects shown in Figure 1. In contrast, Group B (Moderate-Intensity Aerobic Exercises) demonstrated a smaller yet statistically significant improvement, with MoCA scores rising from 25.77±1.45 to 26.71±1.37 ( t=6.02 , df=30 , p=0.0006 ) ( Table 2). The 0.94-point increase did not surpass the MCID, and the marginal reduction in variability (standard deviation: 1.45 to 1.37) suggested heterogeneous responses to aerobic training shown in Figure 2.

**Table 1.** Pre & post Mean score values of MoCA scale within experimental group A.

Test	N	Mean score	Standard deviation	DF	t-value	p-value	Std. Error
Pre	31	25.81	1.14	30	15.87	0.0001	0.28
Post	31	29.13	0.76				

The data in Table 1 show the pre and post values of Montreal Cognitive Assessment (MoCA) scale in experimental group A.

**Table 2.** Pre & post Mean score values of MoCA scale within CONTROL group B.

Test	N	Mean score	Standard deviation	DF	t-value	p-value	Std. Error
Pre	31	25.77	1.45	30	6.10	0.0001	0.15
Post	31	26.71	1.37				

The data in Table 2 show the pre and post test mean values of Montreal Cognitive Assessment (MoCA) scale in CONTROL group B.

**Figure 3.**
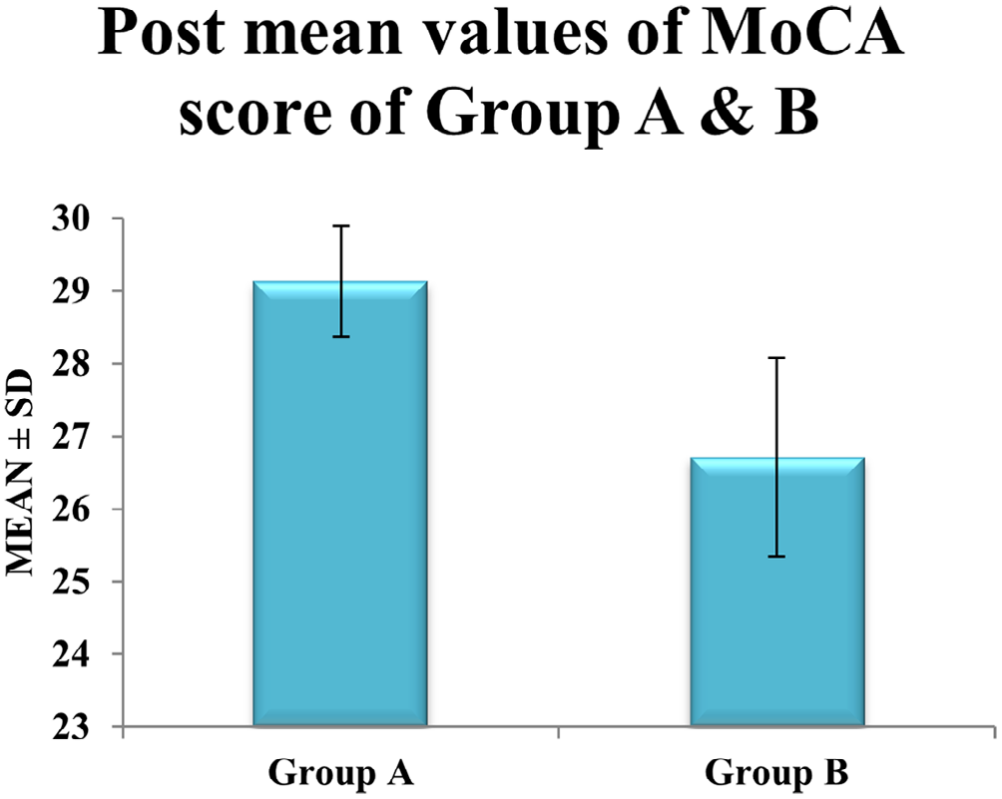
Graphical representation of Means of post values of MoCA between Group A & Group B.

Between-group comparisons further underscored the superiority of blindfolded dual-task training. Post-intervention MoCA scores differed significantly between Group A ( 29.13 ) and Group B ( 26.71 ), with a 2.42-point disparity ( p<0.0001 ) ( Table 3). This finding highlights the added neurocognitive benefits of integrating sensory deprivation into dual-task protocols. Effect size analysis reinforced these results, with Group A demonstrating a large Cohen’s d of 1.89, compared to a moderate effect ( d=0.65 ) in Group B shown in Figure 3. Clinically, the magnitude of improvement in Group A aligns with thresholds linked to reduced dementia risk in longitudinal studies of diabetic populations, emphasizing the potential of blindfolded cognitive-motor interventions to mitigate diabetes-related cognitive decline. These outcomes advocate for the inclusion of sensory-enhanced dual-task training in rehabilitation protocols to optimize cognitive outcomes in type 2 diabetes mellitus.

**Table 3.** Mean score of post-interventional values of MONTREAL COGNITIVE ASSESSMENT (MoCA) between experimental groups A, and control group B.

Test	N	Mean score	Standard deviation	DF	t-value	p-value	Std. Error
Post	31	29.13	0.76	30	8.58	0.0001	0.28
Post	31	26.71	1.37				

The data in Table 3 show the post-mean values of the Montreal Cognitive Assessment Scale for Groups A and B.

## Discussion

The results showed that cognitive motor dual-task training, along with aerobic and resisted exercises, improved significantly ( *p* < 0.0001) the groups’ cognitive scores before and after the tests. A statistically significant difference was observed when these groups were compared. Cognitive motor dual-task training and traditional therapy have different effects on type 2 diabetes mellitus patients’ mental abilities. T2DM patients’ cognitive deterioration is largely due to vascular damage caused by protein accumulation on the blood vessel walls. This process harms endothelial cells, promotes decreased synthesis of vasodilating substances, and generates reactive oxygen species. Consequently, the cerebral blood flow decreases, leading to neurovascular dysfunction and neuronal damage. This pathophysiological mechanism causes an accelerated decline in cognitive domains such as global cognition, processing speed, executive function, verbal fluency, and memory.

Motor cognitive dual-task training (MCDTT) intervention may improve the functional relationship between motor and cognitive brain regions. This type of training enhances gait performance and encourages brain activation. Additionally, it promotes neuroplasticity, which helps patients with neurological abnormalities and motor dysfunctions. Dual-task training is beneficial for walking, a basic motor action that involves the brain, cerebellum, and brainstem and integrates sensory information. The use of blinded training methods improves cognitive processing and interhemispheric communication, further increasing brain activity. By allowing non-visual sensory data to be processed through the visual cortex and promoting neuronal plasticity, sensory substitution strategies are essential. It has been discovered that these modifications are advantageous for both visually impaired individuals and those undergoing cognitive rehabilitation.

Eggenberger et al. (2015) investigated the effect of cognitive training in conjunction with physical activity on cognitive function in older persons. Verbal memory training with treadmill walking (MEMORY), virtual reality-based video game dancing (DANCE), and treadmill walking alone (PHYS) were the three groups of participants aged ≥70 years. Standardized assessments involved The Wechsler Adult Intelligence Scale-Revised (WAIS-R) was used to assess cognitive ability using the Digit Symbol Substitution Task (DSST), Executive Control Task, and Trail Making Test Part B (TMTB). According to their findings, cognitive-physical training considerably improved working memory, executive functioning, and attention switching, especially when used in a dual-task setting. The duration of the intervention also affected how much progress was observed. ^35^

The effects of dual-task training on gait performance of 65-year-olds with type 2 diabetes or older were also examined by Hewston et al. (2013). Participants were assigned to either the healthy control group (HC) or diabetes group (T2DM). Participants in the study completed a 6-meter path at their maximum and preferred walking speeds while completing a motor task (carrying a basket) or a cognitive task (serial subtraction). They found that the gait speed of people with type 2 diabetes was slower than that of people in good health. According to this study, dual-task training considerably improved gait performance in older adults with diabetes. This may be a useful strategy for enhancing motor function in this population. ^36^

The current investigation assessed the impact of the CMDBT in conjunction with aerobic workouts on cognitive function and muscle strength in individuals with T2DM. The Montreal Cognitive Assessment (MoCA) scores showed a statistically significant improvement. the 30-minute CMDBT sessions, which were followed by 30-minute moderate-intensity aerobic and resistance training, were economical and therapist-friendly. It is a good choice for inpatient rehabilitation settings because it is organized and time efficient. Additionally, according to the post-test results, Group A (CMDBT) showed more cognitive improvements than Group B (conventional therapy alone). This demonstrates CMDBT’s potential of the CMDBT as a successful cognitive rehabilitation intervention for patients with type 2 diabetes. Given these results, CMDBT should be used in clinical settings and in future studies to maximize its use in metabolic disorders and neurorehabilitation and further investigate its therapeutic advantages.

## Conclusion

According to this study, 12 weeks of cognitive motor dual-task training produced substantial improvements in cognitive function in patients with type 2 diabetes. The results showed that cognitive motor dual-task training and aerobic exercises significantly increased post-interventional values within the groups; however, when group comparisons were made, cognitive motor dual-task training showed a statistically significant improvement in cognitive function compared to moderate-intensity aerobic exercises. The present findings provide a strong foundation for further studies and clinical implementation of CMDTT in improving cognitive T2DM patients’ function and general quality of life.

### Limitations of the study

• The study includes small sample size, the study did not include long term follow up.
• This study sample size was relatively small to detect the effects of cognitive motor dual-task training (CMDTT) on cognitive function in patients with type 2 diabetes mellitus.

### Recommandations of the study

• Follow-up programs can be included to assess the short- and long-term effects of the treatment.
• Further studies should be conducted to evaluate the effects of cognitive motor dual-task training in other conditions.
• The effects of cognitive motor dual-task training on other types of diabetes and its complications should be studied.
• Further study should include more measurement tools like fMRI.

## Ethics and consent statement

This study was conducted in accordance with the Declaration of Helsinki and was approved by an institutional ethics committee on 30-12-2023 at the Apollo Institute of Medical Science and Research, Chittoor, Andhra Pradesh, India. Ethics Committee Number: **PG/35/IEC/AIMSR/2023.** Written informed consent was obtained from all participants. The study conducted as per guideline of Declaration of Helsinki.

**Table:** DATA COLLECTION SHEET: the study participants are De identified with the serial number

EXPERIMENTAL GROUP 1 - CMDBT			CONTROL GROUP 2 - Conventional Therapy		
S.no	MoCA Score		S.no	MoCA Score	
	Pre-Test	Post-Test		Pre-Test	Post-Test
1	24	28	1	25	26
2	25	29	2	27	28
3	24	29	3	25	26
4	27	30	4	24	25
5	27	30	5	27	25
6	23	28	6	25	27
7	24	29	7	24	24
8	24	28	8	26	27
9	26	30	9	27	28
10	24	29	10	24	25
11	27	29	11	28	29
12	29	30	12	26	28
13	26	29	13	28	28
14	28	30	14	26	27
15	27	30	15	24	27
16	24	28	16	26	27
17	25	29	17	27	28
18	24	28	18	24	26
19	28	30	19	26	26
20	26	29	20	28	28
21	28	30	21	24	25
22	28	30	22	27	28
23	25	29	23	26	26
24	23	28	24	26	27
25	27	30	25	28	29
26	25	29	26	24	25
27	26	29	27	26	27
28	24	28	28	24	26
29	26	29	29	24	25
30	28	29	30	28	29
31	28	30	31	25	26

## Data Availability

The datasets generated analyzed during the current study are available in the Anandh Raj, J (2025). Pretest and post test values of MoCA in Group A and B in Type 2 Diabetes Mellitus subjects. figshare. Dataset. ( https://figshare.com/s/014afef5a58e663a3b96). ^37^ **DOI:** 10.6084/m9.figshare.28513433.V2 The extended data for this study include the demographic dataset of participants have been deposited in the Anandh Raj, J (2025). Baseline characteristics of 12-week & 18th-week follow-up of cognitive motor dual-task training in type 2 diabetes mellitus subjects. figshare. Dataset. https://doi.org/10.6084/m9.figshare.29134604.v1 ^38^ Data are available under the terms of the Creative Commons Attribution 4.0 International license (CC-BY 4.0)
