# The Effect of Visual Deprivation During Cognitive Motor Dual Task Training on Cognitive Function in Type 2 Diabetes Mellitus

**DOI:** 10.12688/f1000research.162466.2

**Published:** 2025-08-19

**Authors:** J Anandh Raj, Ramesh Chandra Patra, Kavitha S, V Subramanyam, K Himabindu, Kilani Kusuma, M.L. Ramya Krishna

**Affiliations:** 1 Lovely Faculty of Applied Medical Sciences, Lovely Professional University, Phagwara, Punjab, 144411, India; 2 Lovely Faculty of Applied Medical Sciences, Lovely Professional University, Phagwara, Punjab, 144411, India; 3 School of Health Sciences, The Apollo University, Chittoor, Andhra Pradesh, 517127, India; 4 School of Health Sciences, The Apollo University, Chittoor, Andhra Pradesh, 517127, India; 5 School of Health Sciences, The Apollo University, Chittoor, Andhra Pradesh, 517127, India; 6 Faculty of Physiotherapy, Meenakshi Academy of Higher Education and Research, Chennai, Tamil Nadu, 600078, India; 7 School of Health Sciences, The Apollo University, Chittoor, Andhra Pradesh, 517127, India

**Keywords:** Type 2 Diabetes mellitus, cognitive function, cognitive motor dual task training, montreal cognitive assessment (MoCA).

## Abstract

**Background:**

Diabetes Mellitus (DM) is a chronic metabolic disorder caused by hyperglycemia, impaired insulin secretion, and insulin resistance. Type 2 diabetes mellitus (T2DM) is associated with an increased risk for cognitive dysfunction. Cognitive motor dual task blindfold training (CMDBT) forces the brain to process motor tasks in one of the four procedural memory centers: the basal ganglia, cerebellum, supplementary motor area, and premotor cortex. Hence, it helps improve cognition in patients with T2DM.

**Methods:**

A randomized controlled trial was conducted with 62 participants diagnosed with type 2 diabetes mellitus. Baseline cognition was assessed using the Montreal Cognitive Assessment (MoCA). The experimental group (n=31) underwent cognitive-motor dual-task blindfold training combined with aerobic and resistance exercises, while the control group (n=31) received conventional aerobic and resistance training only. Both groups participated in their respective interventions three times weekly for 12 weeks. Post-intervention cognitive function was re-assessed using the MoCA scale.

**Results:**

Statistical analysis of the data revealed that there was a significant improvement in cognitive function in experimental group A subjects, with a significant mean difference observed in group A compared to group B. The P value of MoCA was 0.0001 in experimental group A subjects.

**Conclusion:**

CMDBT is more effective than conventional exercise in enhancing cognitive function in individuals with T2DM and may serve as a valuable intervention to mitigate diabetes-associated cognitive decline.

## Introduction

Diabetes mellitus comprises a group of chronic metabolic illnesses distinguished as high blood glucose levels resulting from deficiencies in insulin secretion, insulin action, or both. ^1^ Unlike type 1 diabetes mellitus (T1DM), which is an autoimmune disorder marked by the destruction of insulin-producing beta cells, type 2 diabetes mellitus (T2DM) primarily arises due to insulin resistance along with varying degrees of insulin deficiency. ^2^ The World Health Organization (WHO) estimated that 422 million people worldwide had diabetes in 2014. Nearly four million fatalities annually are attributed to high blood sugar. ^3^ By 2045, there will be at least 629 million diabetics worldwide. As per the International Diabetes Federation (IDF), 850 billion US dollars were spent on adult diabetes-related medical treatment worldwide in 2017. In India, the prevalence of T2DM is rapidly increasing, with significant public health implications. ^4^^,^^5^

Type 2 diabetes is not only a metabolic disorder but also a significant risk factor for cognitive decline and dementia. ^6^^–^^9^ The mechanistic pathways include hyperphosphorylation of tau proteins, which links insulin dysregulation with Alzheimer’s disease pathology. ^10^^–^^13^ Chronic accumulation of advanced glycation end products (AGEs) due to sustained hyperglycemia, ^7^ and endothelial dysfunction causing microvascular damage, neurovascular uncoupling, and reduced cerebral blood flow. ^7^^–^^9^^,^^14^ These pathologies accelerate deterioration in domains such as verbal fluency, executive function, processing speed, memory, and overall cognition. ^14^

Interventions for cognitive impairment in T2DM commonly employ resistance and aerobic training, which improve insulin sensitivity, cardiorespiratory fitness, and hippocampal integrity, thus benefiting cognitive function. ^13^^,^^15^^–^^19^ Multi-modal programs combining physical and cognitive training have demonstrated superior improvements. ^13^^,^^15^^–^^18^^,^^20^ Among these, cognitive-motor dual-task training (CMDT)—involving simultaneous performance of cognitive and motor tasks—capitalizes on synergistic neuroplastic mechanisms, yielding greater cognitive benefits compared to single-modality approaches. ^21^^–^^23^ In CMDT, performing simultaneous cognitive and motor tasks challenges attentional control and postural stability, contributing to improvements in motor-cognitive interference management during walking and balance. ^24^^,^^25^ This aligns with the guided plasticity facilitation framework, which posits that concurrent physical and cognitive stimulation enhances neuroplasticity via factors like increased brain-derived neurotrophic factor (BDNF). ^20^

However, a critical research gap exists regarding the role of visual deprivation during CMDT for T2DM populations. While CMDBT enhances functional connectivity in motor-cognitive brain regions and improves cerebral cortex activation, ^26^^–^^28^ it remains unclear whether removing visual input through blindfolding could further augment neuroplasticity and cognitive outcomes. Visual deprivation via blindfolding functions through sensory substitution, whereby information is rerouted via alternate senses such as tactile and proprioceptive pathways to the visual cortex, promoting cross-modal neuroplasticity. ^29^ Sensory substitution, first introduced by Paul Bach-y-Rita, ^30^ has demonstrated efficacy in cortical reorganization and perceptual compensation particularly among visually impaired individuals. ^31^^,^^32^

Given that individuals with T2DM experience metabolic and microvascular impairments compromising neural plasticity, ^32^^–^^34^ blindfold training may uniquely amplify compensatory brain activation and multisensory integration in this population. By forcing dependence on non-visual sensory modalities, blindfold cognitive-motor dual-task training may serve as a stronger neuroplastic stimulus than training allowing visual input.

The research question for this study is: Does incorporating visual deprivation (blindfolding) into cognitive-motor dual-task training (CMDBT) produce superior improvements in cognitive function among adults with type 2 diabetes mellitus compared to standard conventional therapy involving aerobic and resistance training? The primary aim is to evaluate the effect of cognitive-motor blindfold training on cognitive function in individuals with type 2 diabetes mellitus.

The objective is to compare the effects of CMDBT and standard conventional therapy employing aerobic and resistance training on multiple cognitive domains including memory, executive function, attention, and processing speed, as well as to elucidate neurophysiological mechanisms underlying potential cognitive benefits. Cognitive-Motor Blindfold Training entails simultaneous motor and cognitive tasks performed under enforced visual deprivation via blindfolding. ^30^^–^^32^

Unlike standard conventional therapy, which involves aerobic and resistance training without integrated cognitive tasks or sensory deprivation, CMDBT promotes reliance on proprioceptive, tactile, and vestibular inputs, thereby enhancing multisensory integration and cross-modal plasticity. Physiologically, CMDBT is hypothesized to strengthen alternative neural pathways, stimulate cortical reorganization especially in prefrontal and parietal networks, and increase demands on compensatory processing mechanisms, resulting in superior cognitive resilience. ^32^^–^^34^ This study tested the hypothesis that CMDBT, with its added component of visual deprivation during dual-task training, led to significantly greater improvements in cognitive function than standard conventional therapy of aerobic and resistance training in individuals with T2DM by leveraging enhanced neuroplasticity induced through sensory deprivation and multisensory reweighting.

## Method

Based on the literature, cognitive motor dual-task blindfold training has been applied in cognitive studies, but there is insufficient evidence regarding its use in individuals with type 2 diabetes mellitus (T2DM). This study was approved by the Institutional Ethics Committee of Apollo Institute of Medical Science and Research, Chittoor, Andhra Pradesh, India, on 30-12-2023 (Ethics Committee Number: PG/35/IEC/AIMSR/2023). All participants provided written informed consent prior to enrollment.

**Study design:** This was a single-blinded, randomized controlled trial (RCT) conducted according to CONSORT guidelines (Clinical Trials Registry of India, CTRI/2024/01/061956). The study population included patients diagnosed with T2DM attending the physiotherapy outpatient department at Apollo District Headquarters Hospital, Murukambattu, Chittoor, Andhra Pradesh, India. Participants were selected through purposive sampling to meet inclusion criteria and were randomly allocated to two groups using a lottery method to ensure unbiased assignment.

### Participant flow

**Figure 1.**
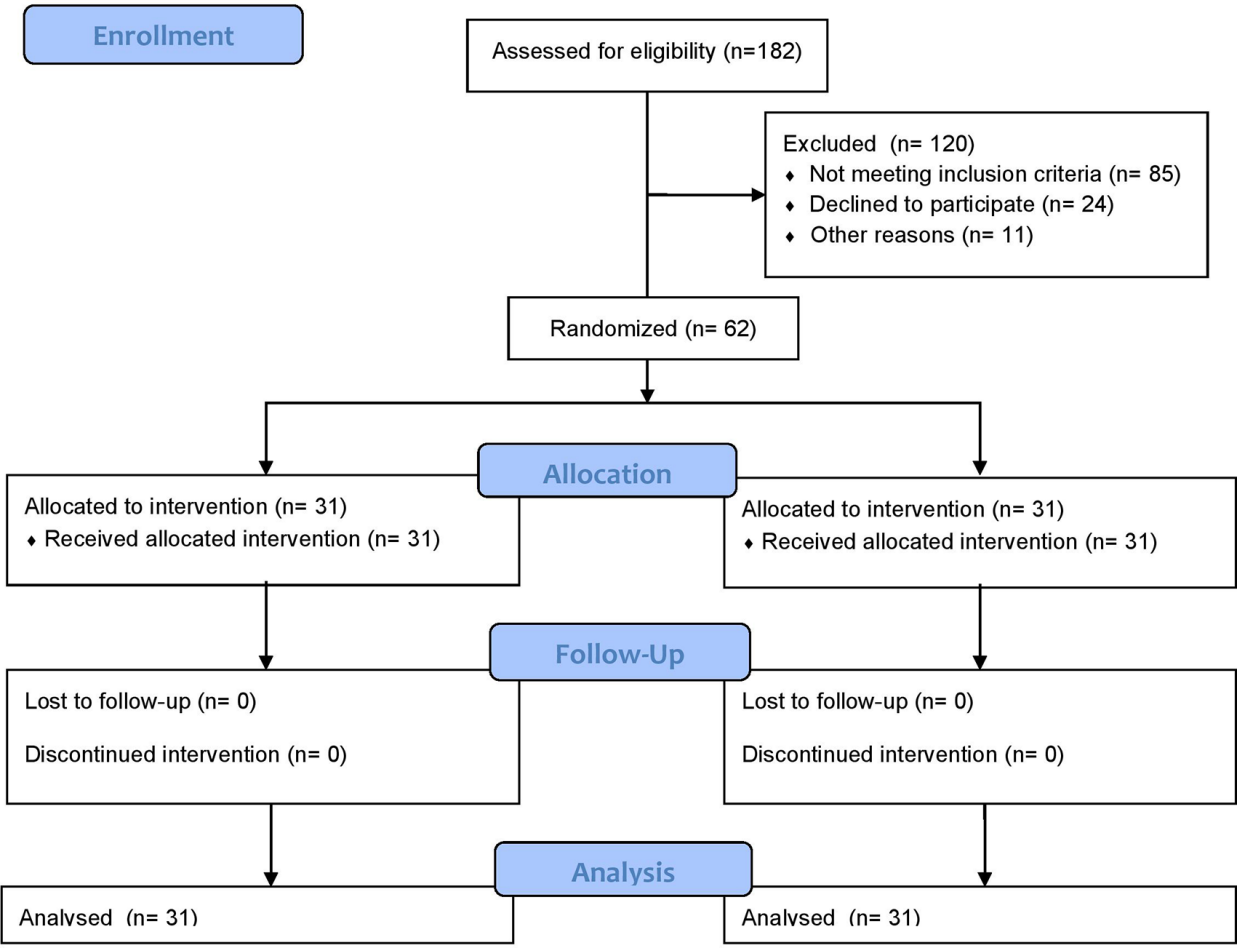
CONSORT flow diagram illustrating the recruitment, randomization, allocation to Cognitive-Motor Dual-Task Blindfold Training (CMDBT) and control (conventional aerobic-resistance therapy) groups, participant follow-up, and inclusion in the analysis of cognitive function outcomes in adults with type 2 diabetes mellitus.

A total of 182 participants were assessed for eligibility. Of these, 120 were excluded due to not meeting inclusion criteria or declining to participate. The remaining 62 eligible participants were randomized equally into two groups: Group A (Cognitive-Motor Dual-Task Blindfold Training, CMDBT; n = 31) and Group B (Control: Moderate-Intensity Aerobic and Resistance Training; n = 31). All participants in both groups received their allocated interventions and completed the study protocol. There were no dropouts and follow-up. Data from all 62 participants were analyzed on an intention-to-treat basis. The CONSORT Flow Diagram illustrating participant enrollment, allocation, follow-up, and analysis in Figure 1.

### Sample size calculation

The sample size for this randomized controlled trial was calculated based on detecting a clinically meaningful difference between two independent groups on the primary outcome measure, the Montreal Cognitive Assessment (MoCA) score. Using data from previous studies on cognitive interventions in type 2 diabetes mellitus populations, an expected mean difference of approximately 2.3 points (the minimal clinically important difference for MoCA) with an estimated standard deviation of 2.0 was assumed. The calculation employed a two-sided independent samples t-test with a significance level (alpha) of 0.05 and desired statistical power of 80% (beta = 0.20) to minimize Type I and Type II errors, respectively. Based on these parameters, the minimum required sample size was estimated at 31 participants per group. This sample size also accounted for ensuring sufficient power to detect meaningful cognitive improvements while considering practical constraints in recruitment. Allocation concealment and randomization were designed accordingly to maintain balance across groups.

**Randomization:** A total of 62 participants were enrolled and randomly assigned equally into two groups (31 per group) using a lottery method. Allocation concealment was maintained through sequentially numbered, sealed, opaque envelopes prepared by an independent investigator uninvolved in assessment.

**Blinding:** Participants were blinded to the group allocation to reduce performance bias. Outcome assessors and data analysts were also blinded to ensure unbiased evaluation of results.

**Inclusion criteria:**

• Diagnosed with T2DM
• HbA1c values >6.5 mmol/dL
• Education level exceeding 5 years (ability to read and write)
• Diabetes duration between 5 and 10 years
• Both male and female subjects
• Willingness to participate and signed informed consent

**Exclusion criteria:**

• Refusal or inability to cooperate
• Musculoskeletal anomalies
• Presence of pressure sores or ulcers
• Exposure to radiological or X-ray therapy within the past 6 months
• Microvascular circulation defects
• Diabetic neuropathy
• Unstable vital signs
• Cardiac anomalies
• Malignant tumors

### Interventions

In this study, participants who fulfilled the selection criteria were asked to provide written informed consent. Baseline measurements were obtained after obtaining consent. A total of 62 subjects were allotted randomly to group A (experimental) and group B (control) in 1:1 parallel with the lottery method. The subjects who fulfilled the eligibility criteria underwent pre interventional assessment MoCA.

### Experimental group

Participants underwent a structured, multicomponent training regimen thrice weekly for 12 weeks (36 sessions total). Each session integrated progressive cognitive motor dual-task exercises performed under blindfolded conditions, using a treadmill to enhance motor control challenges while limiting visual input. Cognitive Training: Tasks targeting working memory (digit span, word list recall), visuospatial skills (auditory clock interpretation), executive function (serial arithmetic, verbal sequencing), attention (digit ordering, auditory detection), and language processing (verbal memory and fluency). Task difficulty progressively increased every four weeks to optimize neuroplastic adaptation. Each session included 10 cognitive-motor dual-task trials. Along with CMDBT all the subjects received standard conventional therapy of Moderate-intensity resistance exercises (50–69% one-repetition maximum) using resistance bands for major joints (shoulder, elbow, wrist, hip, knee, ankle) with 10 repetitions per exercise. Aerobic training at 55–70% maximum heart rate via cycling and treadmill walking. Rest breaks were allowed as needed for participant comfort.

### Control group

Participants received standard conventional physiotherapy including moderate-intensity aerobic and resistance training thrice weekly for 12 weeks (36 sessions total)—mirroring the frequency and intensity of the experimental group but without cognitive or blindfold components. Resistance exercises targeting the same muscle groups with matched intensity and repetitions as the experimental group. Aerobic training at 55–70% maximum heart rate using cycling and treadmill.

### Outcome measures

The primary outcome was cognitive function measured by the Montreal Cognitive Assessment (MoCA) at baseline of 0 ^th^ week and post-intervention at 12 ^th^ week.

### Interim analysis and safety monitoring

Participant adherence, safety, and adverse events were monitored continuously, with interim analyses conducted monthly to evaluate compliance, efficacy signals, and participant well-being.

### Statistical analysis

Statistical analysis was performed using IBM SPSS Statistics 30 version ^19^ under subscription version. with a two-tailed alpha level of 0.05 defining significance. Normality of data distribution was confirmed via Shapiro-Wilk tests ( *W* > 0.90 for all groups). Within-group changes in MoCA scores were analyzed using paired *t*-tests, while between-group differences at post-intervention were assessed via independent *t*-tests. Effect sizes were calculated using Cohen’s *d*, interpreted as small ( *d* = 0.20), medium ( *d* = 0.50), and large ( *d* ≥ 0.80). Homogeneity of variance was verified with Levene’s test ( *p* > 0.10 for all comparisons), supporting the use of equal variances assumed in *t*-tests. Clinical significance was evaluated against the established minimal clinically important difference (MCID) of 2.3 points for MoCA in diabetic populations. All data are reported as mean ± standard deviation (SD), with 95% confidence intervals (CI) calculated for mean differences.

## Results

A total of 62 participants diagnosed with type 2 diabetes mellitus were randomly allocated into two groups: Group A (Cognitive-Motor Dual-Task Blindfold Training, CMDBT; n = 31) and Group B (Control: Moderate-Intensity Aerobic and Resistance Training; n = 31). Baseline demographic and clinical characteristics were comparable between groups, with no statistically significant differences observed in age, gender distribution, duration of diabetes, educational level, HbA1c, body mass index (BMI), or Mini-Mental State Examination (MMSE) scores (all p > 0.05), confirming homogeneity. ^37^ The normality of Montreal Cognitive Assessment (MoCA) scores was verified using the Shapiro-Wilk test (p > 0.05), allowing the use of parametric statistical analyses. There were no dropouts during the intervention period, and all participants completed baseline and post-intervention assessments. Data were analyzed on an intention-to-treat basis.

**Figure 2.**
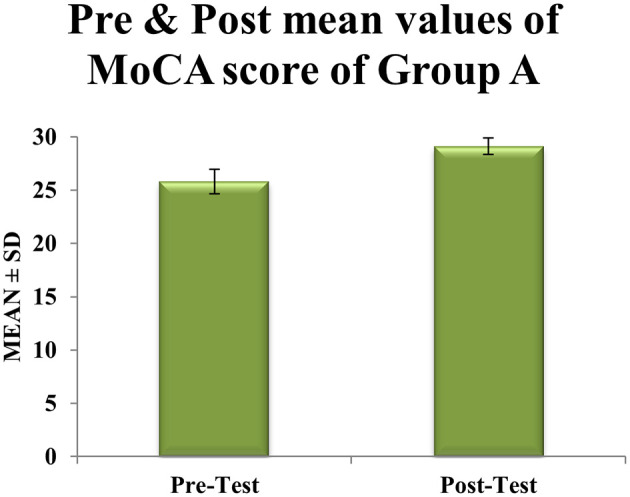
Graphical representation of Means of pre and post values of MoCA within experimental Group A.

**Figure 3.**
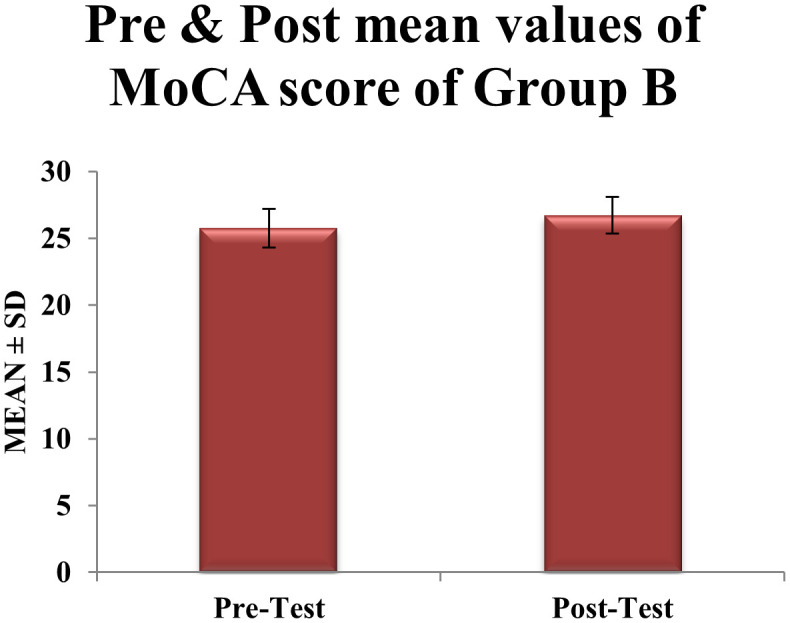
Graphical representation of Means of pre and post values of MoCA within CONTROL Group B.

Within-group analyses showed significant improvements in cognitive function for both groups. In Group A (CMDBT), mean MoCA scores increased by 3.32 points, from 25.81 ± 1.74 pre-intervention to 29.13 ± 0.76 post-intervention (t = 6.32, df = 30, p < 0.0001). This increase exceeded the minimal clinically important difference (MCID) of 2.3 points and was accompanied by a 56% reduction in score variability (SD reduced from 1.74 to 0.76), indicating a consistent and robust treatment effect ( Table 1, Figure 2). In contrast, **Group B (control)** exhibited a statistically significant but smaller mean increase of **0.94 points** in MoCA scores, from 25.77 ± 1.45 to 26.71 ± 1.37 (t = 6.02, df = 30, p = 0.0006). However, this gain did not surpass the MCID threshold. The standard deviation decreased marginally by about 5.5% (from 1.45 to 1.37), suggesting more variability in response to aerobic and resistance training alone ( Table 2, Figure 3).

**Table 1.** Pre & post Mean score values of MoCA scale within experimental group A.

Test	N	Mean score	Standard deviation	DF	t-value	p-value	Std. Error
Pre	31	25.81	1.14	30	15.87	0.0001	0.28
Post	31	29.13	0.76				

The data in Table 1 show the pre and post values of Montreal Cognitive Assessment (MoCA) scale in experimental group A.

**Table 2.** Pre & post Mean score values of MoCA scale within CONTROL group B.

Test	N	Mean score	Standard deviation	DF	t-value	p-value	Std. Error
Pre	31	25.77	1.45	30	6.10	0.0001	0.15
Post	31	26.71	1.37				

The data in Table 2 show the pre and post test mean values of Montreal Cognitive Assessment (MoCA) scale in CONTROL group B.

**Figure 4.**
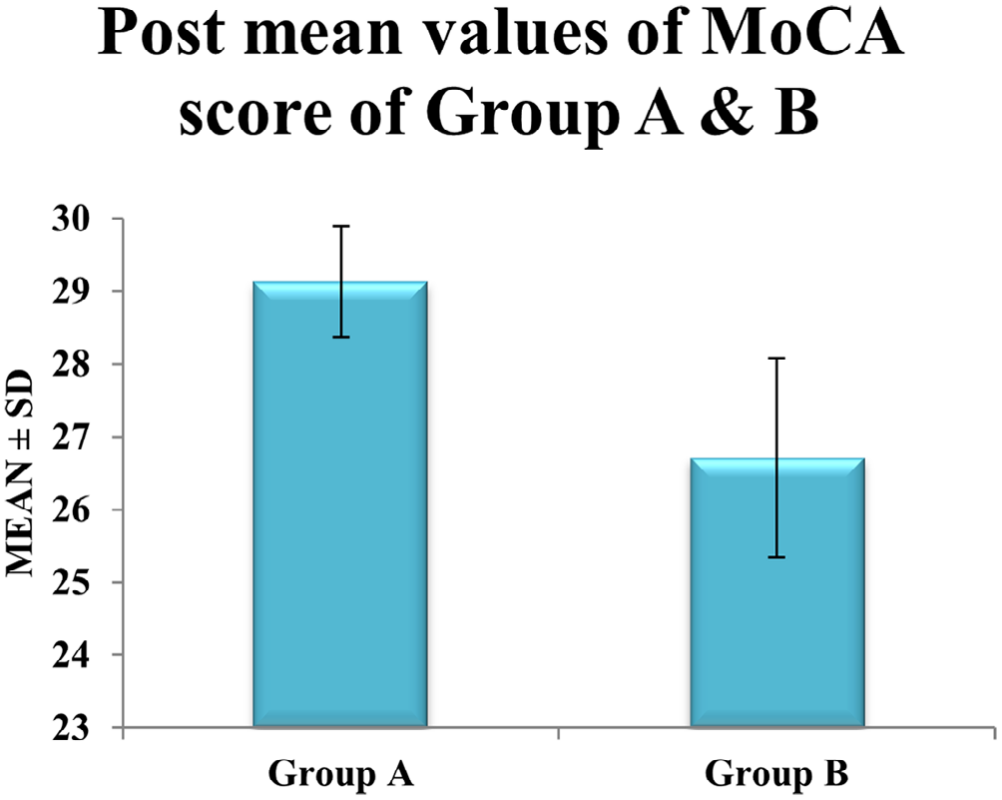
Graphical representation of Means of post values of MoCA between Group A & Group B.

Between-group comparison demonstrated that **post-intervention MoCA scores in Group A (29.13 ± 0.76) were significantly higher than in Group B (26.71 ± 1.37)**, with a mean difference of **2.42 points** (p < 0.0001) (see Table 3). Effect size analysis showed a large effect for Group A (Cohen’s d = 1.89) versus a moderate effect for Group B (d = 0.65) ( Figure 3), further supporting the superior cognitive benefits of the blindfolded cognitive-motor dual-task training ( Table 3, Figure 4).

**Table 3.** Mean score of post-interventional values of MONTREAL COGNITIVE ASSESSMENT (MoCA) between experimental groups A, and control group B.

Test	N	Mean score	Standard deviation	DF	t-value	p-value	Std. Error
Post	31	29.13	0.76	30	8.58	0.0001	0.28
Post	31	26.71	1.37				

The data in Table 3 show the post-mean values of the Montreal Cognitive Assessment Scale for Groups A and B.

Clinically, the magnitude of improvement in Group A aligns with thresholds associated with reduced dementia risk in diabetic populations, highlighting the potential of integrating sensory deprivation to augment cognitive rehabilitation in type 2 diabetes mellitus.

These findings advocate for incorporating sensory-enhanced cognitive-motor dual-task training into rehabilitation protocols to optimize cognitive outcomes and potentially mitigate diabetes-associated cognitive decline.

## Discussion

This study demonstrated that cognitive-motor dual-task blindfold training (CMDBT) combined with moderate-intensity aerobic and resistance exercises significantly improved cognitive function in individuals with type 2 diabetes mellitus (T2DM). Both intervention groups exhibited statistically significant cognitive gains before and after intervention (p < 0.0001), but participants receiving CMDBT showed significantly greater improvement compared to those undergoing conventional therapy alone. These results highlight the differential impact of multimodal cognitive-motor interventions versus standard exercise protocols on cognitive outcomes in T2DM.

Cognitive decline in T2DM is multifactorial, with vascular dysfunction playing a critical role. Elevated blood glucose leads to protein accumulation on vascular walls, causing damage to endothelial cells and reducing synthesis of vasodilating substances like nitric oxide, while promoting reactive oxygen species generation and oxidative stress. This pathophysiological cascade impairs cerebral blood flow, resulting in neurovascular uncoupling and neuronal injury. Such mechanisms accelerate deterioration in cognitive domains including global cognition, executive function, processing speed, verbal fluency, and memory. This mechanistic explanation is supported by recent literature and clearly distinguishes pathological processes from study results.

The cognitive benefits observed in the CMDBT group are likely attributable to enhanced functional connectivity between motor and cognitive brain regions induced by dual-task training. Such training has the potential to improve gait and other motor functions while simultaneously promoting brain activation and neuroplasticity through complex neural circuit engagement. Walking, as a motor behavior, integrates sensory inputs processed by the brain, cerebellum, and brainstem, making it an ideal target for dual-task interventions. The addition of blindfolding enforces sensory substitution by rerouting non-visual information through the visual cortex, thereby enhancing cross-modal neuronal plasticity and interhemispheric communication. These mechanisms align with findings from sensory substitution research demonstrating cortical reorganization beneficial in both visually impaired individuals and those undergoing cognitive rehabilitation.

Eggenberger et al. (2015) studied older adults (≥70 years) using combined cognitive and physical training modalities, including verbal memory tasks paired with treadmill walking and virtual reality dancing. ^35^ Utilizing standardized cognitive assessments such as the Digit Symbol Substitution Task and Trail Making Test Part B, they found that cognitive-physical dual-task training significantly improved working memory, executive functioning, and attention switching—especially with longer intervention durations. Our findings align with Eggenberger et al., reinforcing the concept that simultaneous cognitive and physical tasks can confer additive cognitive benefits in populations at risk for decline. ^35^

Similarly, Hewston et al. (2013) investigated dual-task effects on gait performance in adults aged 65 and older with T2DM, reporting slower gait speeds in diabetic participants compared to healthy controls. ^36^ Their findings showed that dual-task training improved gait performance in diabetics, suggesting enhanced motor function. Although Hewston et al. focused primarily on motor outcomes, their results provide indirect support for the neurological benefit of dual-task approaches, consistent with the cognitive improvements observed here with CMDBT. The superior cognitive improvements demonstrated by the CMDBT group in this study are consistent with previous literature on multimodal and dual-task interventions. Notably, Eggenberger et al. (2015) ^35^ found that multicomponent physical exercise programs incorporating simultaneous cognitive training yielded significantly greater cognitive benefits for older adults compared to programs that included only physical exercise. Similarly, Hewston and Deshpande (2013) ^36^ reported that dual-task balance training improved gait parameters and reduced cognitive-motor interference among older adults with type 2 diabetes mellitus. These findings complement the current study by emphasizing the value of interventions that actively engage both cognitive and motor domains to enhance neuroplasticity and functional outcomes in clinical populations. Our results expand on this evidence, further suggesting that integrating visual deprivation into cognitive-motor dual-task training may amplify neuroplasticity and cognitive resilience, particularly in those with T2DM, by stimulating compensatory and multisensory neural pathways.

This study advances the literature by incorporating blindfold-induced sensory deprivation into cognitive-motor dual-task training, a novel approach that appears to potentiate neuroplasticity beyond conventional dual-task protocols. The structured 30-minute CMDBT sessions, combined with 30 minutes of moderate-intensity aerobic and resistance training, demonstrated feasibility, time-efficiency, and cost-effectiveness, making it well-suited for inpatient rehabilitation settings. Compared to conventional therapy, the significantly superior cognitive gains observed in the CMDBT group emphasize the value of sensory substitution strategies as critical adjuncts to optimize cognitive rehabilitation in T2DM.

Given these findings, we strongly recommend including CMDBT in clinical practice for managing cognitive decline associated with type 2 diabetes. Future research should explore long-term cognitive and functional outcomes, dose-response relationships, and underlying neurophysiological mechanisms using modalities such as neuroimaging or biomarker analyses.

## Conclusion

This study demonstrated that a 12-week cognitive-motor dual-task training (CMDBT) program, combined with aerobic and resistance exercises, produced significant and clinically meaningful improvements in cognitive function among individuals with type 2 diabetes mellitus (T2DM). While both CMDBT and moderate-intensity aerobic exercise interventions yielded significant within-group cognitive gains, CMDBT resulted in significantly greater enhancements compared to aerobic training alone. These findings highlight CMDBT’s potential as an effective, feasible, and time-efficient intervention to mitigate cognitive decline associated with T2DM. The robust improvements observed provide a strong rationale for integrating CMDBT into clinical rehabilitation protocols and warrant further research to explore its long-term benefits and underlying neurophysiological mechanisms. Ultimately, such interventions may contribute to improving cognitive health and overall quality of life in patients living with T2DM.

### Limitations of the study

• The study includes small sample size, the study did not include long term follow up.
• This study sample size was relatively small to detect the effects of cognitive motor dual-task training (CMDTT) on cognitive function in patients with type 2 diabetes mellitus.

### Recommandations of the study

• Follow-up programs can be included to assess the short- and long-term effects of the treatment.
• Further studies should be conducted to evaluate the effects of cognitive motor dual-task training in other conditions.
• The effects of cognitive motor dual-task training on other types of diabetes and its complications should be studied.
• Further study should include more measurement tools like fMRI.

## Ethics and consent statement

This study was conducted in accordance with the Declaration of Helsinki and was approved by an institutional ethics committee on 30-12-2023 at the Apollo Institute of Medical Science and Research, Chittoor, Andhra Pradesh, India. Ethics Committee Number: **PG/35/IEC/AIMSR/2023.** Written informed consent was obtained from all participants. The study conducted as per guideline of Declaration of Helsinki.

**Table:** DATA COLLECTION SHEET: the study participants are De identified with the serial number

EXPERIMENTAL GROUP A - CMDBT			CONTROL GROUP B - Conventional Therapy		
S.no	MoCA Score		S.no	MoCA Score	
	Pre-Test	Post-Test		Pre-Test	Post-Test
1	24	28	1	25	26
2	25	29	2	27	28
3	24	29	3	25	26
4	27	30	4	24	25
5	27	30	5	27	25
6	23	28	6	25	27
7	24	29	7	24	24
8	24	28	8	26	27
9	26	30	9	27	28
10	24	29	10	24	25
11	27	29	11	28	29
12	29	30	12	26	28
13	26	29	13	28	28
14	28	30	14	26	27
15	27	30	15	24	27
16	24	28	16	26	27
17	25	29	17	27	28
18	24	28	18	24	26
19	28	30	19	26	26
20	26	29	20	28	28
21	28	30	21	24	25
22	28	30	22	27	28
23	25	29	23	26	26
24	23	28	24	26	27
25	27	30	25	28	29
26	25	29	26	24	25
27	26	29	27	26	27
28	24	28	28	24	26
29	26	29	29	24	25
30	28	29	30	28	29
31	28	30	31	25	26

## Data Availability

The datasets generated analyzed during the current study are available in the Anandh Raj, J (2025). Pretest and post test values of MoCA in Group A and B in Type 2 Diabetes Mellitus subjects. figshare. Dataset. ( https://figshare.com/s/014afef5a58e663a3b96). ^37^ **DOI:** 10.6084/m9.figshare.28513433.V2 The extended data for this study include the demographic dataset of participants have been deposited in the Anandh Raj, J (2025). Baseline characteristics of 12-week & 18th-week follow-up of cognitive motor dual-task training in type 2 diabetes mellitus subjects. figshare. Dataset. https://doi.org/10.6084/m9.figshare.29134604.v1 ^38^ Data are available under the terms of the Creative Commons Attribution 4.0 International license (CC-BY 4.0)
