# The Effect Of Visual Deprivation During Cognitive Motor Dual Task Training On Cognitive Function In Type 2 Diabetes Mellitus

**DOI:** 10.12688/f1000research.162466.3

**Published:** 2025-10-17

**Authors:** J Anandh Raj, Ramesh Chandra Patra, Kavitha S, V Subramanyam, K Himabindu, Kilani Kusuma, M.L. Ramya Krishna

**Affiliations:** 1 Lovely Faculty of Applied Medical Sciences, Lovely Professional University, Phagwara, Punjab, 144411, India; 2 Lovely Faculty of Applied Medical Sciences, Lovely Professional University, Phagwara, Punjab, 144411, India; 3 School of Health Sciences, The Apollo University, Chittoor, Andhra Pradesh, 517127, India; 4 School of Health Sciences, The Apollo University, Chittoor, Andhra Pradesh, 517127, India; 5 School of Health Sciences, The Apollo University, Chittoor, Andhra Pradesh, 517127, India; 6 Faculty of Physiotherapy, Meenakshi Academy of Higher Education and Research, Chennai, Tamil Nadu, 600078, India; 7 School of Health Sciences, The Apollo University, Chittoor, Andhra Pradesh, 517127, India

**Keywords:** Type 2 Diabetes mellitus, cognitive function, cognitive motor dual task training, montreal cognitive assessment (MoCA).

## Abstract

**Background:**

Type 2 diabetes mellitus (T2DM) is linked to cognitive dysfunction, and cognitive motor dual-task blindfold training (CMDBT) may enhance cognition by engaging procedural memory centers. This study aimed to evaluate the effectiveness of CMDBT combined with aerobic and resistance exercises on cognitive function in individuals with T2DM.

**Methods:**

A single-center, parallel-group randomized controlled trial with pre- and post-intervention assessments was conducted. Sixty two adults with T2DM were randomly allocated to an experimental group (CMDBT plus moderate-intensity aerobic and resistance training; n = 31) or a control group (aerobic and resistance training only; n = 31). Both groups trained three times per week for 12 weeks. Cognitive function was assessed using the Montreal Cognitive Assessment (MoCA) at baseline and after the 12-week intervention.

**Results:**

Baseline demographic and clinical characteristics were comparable between groups (all p > 0.05). After 12 weeks, the experimental group showed a mean MoCA increase of 3.32 ± 1.74 to 29.13 ± 0.76 (p < 0.0001), exceeding the minimal clinically important difference of 2.3 points, whereas the control group improved by 0.94 points (25.77 ± 1.45 to 26.71 ± 1.37; p = 0.0006). The adjusted between-group difference was 2.38 points (p = 0.0001), demonstrating a significantly greater cognitive benefit with CMDBT.

**Conclusion:**

CMDBT combined with conventional exercises is more effective than CMDT or conventional therapy alone in enhancing cognition and in T2DM, supporting its integration into rehabilitation programs.

## Introduction

Diabetes mellitus comprises a group of chronic metabolic illnesses distinguished as high blood glucose levels resulting from deficiencies in insulin secretion, insulin action, or both. ^1^ Unlike type 1 diabetes mellitus (T1DM), which is an autoimmune disorder marked by the destruction of insulin-producing beta cells, type 2 diabetes mellitus (T2DM) primarily arises due to insulin resistance along with varying degrees of insulin deficiency. ^2^ The World Health Organization (WHO) estimated that 422 million people worldwide had diabetes in 2014. Nearly four million fatalities annually are attributed to high blood sugar. ^3^ By 2045, there will be at least 629 million diabetics worldwide. According to the latest 2025 International Diabetes Federation Diabetes Atlas, approximately 589 million adults aged 20 to 79 worldwide currently live with diabetes, with projections rising to over 850 million by 2050. ^4^ The India has one of the highest burdens of diabetes worldwide, with approximately 101 million adults aged 20-79 living with diabetes as of 2025. In India, the prevalence of T2DM is rapidly increasing, with significant public health implications. ^5^

Type 2 diabetes is not only a metabolic disorder but also a significant risk factor for cognitive decline and dementia. ^6^^–^^9^ The mechanistic pathways include hyperphosphorylation of tau proteins, which links insulin dysregulation with Alzheimer’s disease pathology. ^10^^–^^13^ Chronic accumulation of advanced glycation end products (AGEs) due to sustained hyperglycemia, ^7^ and endothelial dysfunction causing microvascular damage, neurovascular uncoupling, and reduced cerebral blood flow. ^7^^–^^9^^,^^14^ These pathologies accelerate deterioration in domains such as verbal fluency, executive function, processing speed, memory, and overall cognition. ^14^

Interventions for cognitive impairment in T2DM commonly employ resistance and aerobic training, which improve insulin sensitivity, cardiorespiratory fitness, and hippocampal integrity, thus benefiting cognitive function. ^13^^,^^15^^–^^19^ Multi-modal programs combining physical and cognitive training have demonstrated superior improvements. ^13^^,^^15^^–^^18^^,^^20^ Among these, cognitive-motor dual-task training (CMDT)—involving simultaneous performance of cognitive and motor tasks—capitalizes on synergistic neuroplastic mechanisms, yielding greater cognitive benefits compared to single-modality approaches. ^21^^–^^23^ In CMDT, performing simultaneous cognitive and motor tasks challenges attentional control and postural stability, contributing to improvements in motor-cognitive interference management during walking and balance. ^24^^,^^25^ This aligns with the guided plasticity facilitation framework, which posits that concurrent physical and cognitive stimulation enhances neuroplasticity via factors like increased brain-derived neurotrophic factor (BDNF). ^20^

However, a critical research gap exists regarding the role of visual deprivation during CMDT for T2DM populations. While cognitive-motor dual-task blindfold training (CMDBT) enhances functional connectivity in motor-cognitive brain regions and improves cerebral cortex activation, ^26^^–^^28^ it remains unclear whether removing visual input through blindfolding could further augment neuroplasticity and cognitive outcomes. Visual deprivation via blindfolding functions through sensory substitution, whereby information is rerouted via alternate senses such as tactile and proprioceptive pathways to the visual cortex, promoting cross-modal neuroplasticity. ^29^ Sensory substitution, first introduced by Paul Bach-y-Rita, ^30^ has demonstrated efficacy in cortical reorganization and perceptual compensation particularly among visually impaired individuals. ^31^^,^^32^

Given that individuals with T2DM experience metabolic and microvascular impairments compromising neural plasticity, ^32^^–^^34^ combined cognitive and physical training modalities, including verbal memory tasks paired with treadmill walking and virtual reality dancing. Utilizing standardized cognitive assessments such as the Digit Symbol Substitution Task and Trail Making Test Part B, they found that cognitive-physical dual-task training significantly improved working memory, executive functioning, and attention switching especially with longer intervention durations. ^35^^,^^36^ Building on these findings, the blindfold training may uniquely amplify compensatory brain activation and multisensory integration in this population. By forcing dependence on non-visual sensory modalities, blindfold cognitive-motor dual-task training may serve as a stronger neuroplastic stimulus than training allowing visual input. ^37^

The research question for this study is: Does incorporating visual deprivation into cognitive-motor dual-task training produce superior improvements in cognitive function among adults with T2DM compared to standard conventional therapy involving aerobic and resistance training? The primary aim is to evaluate the effect of cognitive-motor blindfold training on cognitive function in individuals with type 2 diabetes mellitus.

The objective is to compare the effects of CMDBT and standard conventional therapy employing aerobic and resistance training on cognition function in type 2 diabetes mellitus.

Cognitive-Motor Blindfold Training entails simultaneous motor and cognitive tasks performed under enforced visual deprivation via blindfolding. ^30^^–^^32^

Unlike standard conventional therapy, which involves aerobic and resistance training without integrated cognitive tasks or sensory deprivation, CMDBT promotes reliance on proprioceptive, tactile, and vestibular inputs, thereby enhancing multisensory integration and cross-modal plasticity. Physiologically, CMDBT is hypothesized to strengthen alternative neural pathways, stimulate cortical reorganization especially in prefrontal and parietal networks, and increase demands on compensatory processing mechanisms, resulting in superior cognitive resilience. ^32^^–^^34^ This study tested the hypothesis that CMDBT led to significantly greater improvements in cognitive function than standard conventional therapy of aerobic and resistance training in individuals with T2DM by leveraging enhanced neuroplasticity induced through sensory deprivation and multisensory reweighting.

## Method

### Study design and ethical approval

This single-blinded, randomized controlled trial (RCT) was conducted according to CONSORT guidelines (Clinical Trials Registry of India, CTRI/2024/01/061956). Ethical approval was obtained from the Institutional Ethics Committee of Apollo Institute of Medical Science and Research, Chittoor, Andhra Pradesh, India (Ethics Committee Number: PG/35/IEC/AIMSR/2023) on 30-12-2023. All participants provided written informed consent prior to enrollment.

#### Participant flow

**Figure 1.**
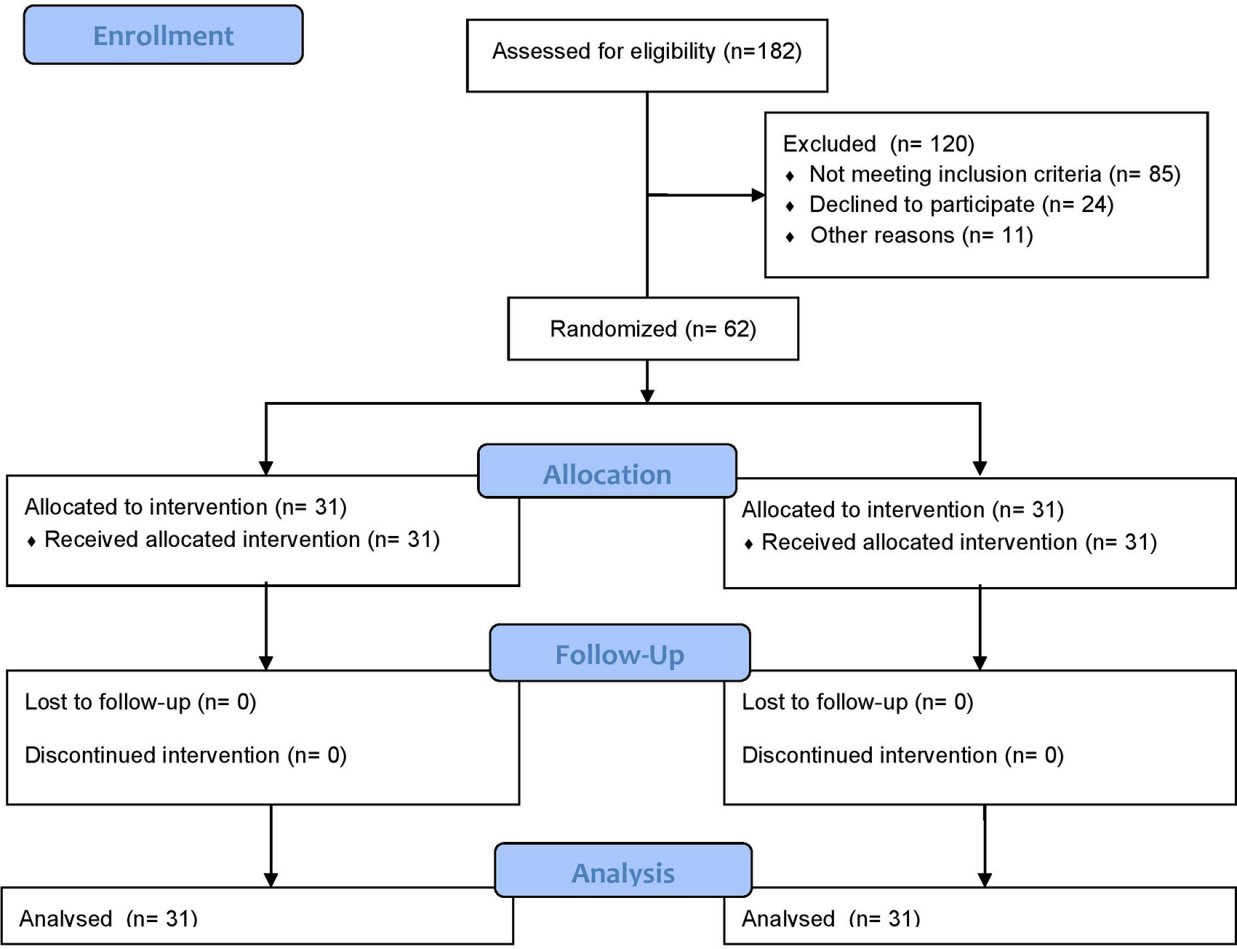
CONSORT flow diagram illustrating the recruitment, randomization, allocation to Cognitive-Motor Dual-Task Blindfold Training (CMDBT) and control (conventional aerobic-resistance therapy) groups, participant follow-up, and inclusion in the analysis of cognitive function outcomes in adults with type 2 diabetes mellitus.

A total of 182 participants were assessed for eligibility. Of these, 120 were excluded due to not meeting inclusion criteria or declining to participate. The remaining 62 eligible participants were randomized equally into two groups: Group A (Cognitive-Motor Dual-Task Blindfold Training, CMDBT; n = 31) and Group B (Control: Moderate-Intensity Aerobic and Resistance Training; n = 31). All participants in both groups received their allocated interventions and completed the study protocol. There were no dropouts and follow-up. Data from all 62 participants were analyzed on an intention-to-treat basis. The CONSORT Flow Diagram illustrating participant enrollment, allocation, follow-up, and analysis in Figure 1.

#### Sample size calculation

The sample size for this randomized controlled trial was calculated based on detecting a clinically meaningful difference between two independent groups on the primary outcome measure, the Montreal Cognitive Assessment (MoCA) score. Using data from previous studies on cognitive interventions in type 2 diabetes mellitus populations, an expected mean difference of approximately 2.3 points (the minimal clinically important difference for MoCA) with an estimated standard deviation of 2.0 was assumed. ^38, 39^ The calculation employed a two-sided independent samples t-test with a significance level (alpha) of 0.05 and desired statistical power of 80% (beta = 0.20) ^40^ to minimize Type I and Type II errors, respectively. Based on these parameters, the minimum required sample size was estimated at 31 participants per group. This sample size also accounted for ensuring sufficient power to detect meaningful cognitive improvements while considering practical constraints in recruitment. Allocation concealment and randomization were designed accordingly to maintain balance across groups.

**Randomization:** A total of 62 participants were enrolled and randomly assigned equally into two groups (31 per group) using a lottery method. Allocation concealment was maintained through sequentially numbered, sealed, opaque envelopes prepared by an independent investigator uninvolved in assessment.

**Blinding:** Participants were blinded to the group allocation to reduce performance bias. Outcome assessors and data analysts were also blinded to ensure unbiased evaluation of results.

**Inclusion criteria:**

- Diagnosed with T2DM
- HbA1c values >6.5 mmol/dL
- Education level exceeding 5 years (ability to read and write)
- Diabetes duration between 5 and 10 years
- Both male and female subjects
- Willingness to participate and signed informed consent

**Exclusion criteria:**

- Refusal or inability to cooperate
- Musculoskeletal anomalies
- Presence of pressure sores or ulcers
- Exposure to radiological or X-ray therapy within the past 6 months
- Microvascular circulation defects
- Diabetic neuropathy
- Unstable vital signs
- Cardiac anomalies
- Malignant tumors, Participants engaging in high levels of habitual physical activity were excluded to minimize confounding effects on cognitive outcomes and ensure homogeneity between groups.

### Interventions

In this study, participants who fulfilled the selection criteria were asked to provide written informed consent. Baseline measurements were obtained after obtaining consent. A total of 62 subjects were allotted randomly to group A (experimental) and group B (control) in 1:1 parallel with the lottery method. The subjects who fulfilled the eligibility criteria underwent pre interventional assessment MoCA.

### Experimental group

Participants completed a structured, multicomponent program three times per week for 12 weeks (36 sessions). Each session combined progressive cognitive–motor dual-task exercises performed under blindfolded conditions while walking on a treadmill to increase motor-control demands and reduce visual input. Cognitive tasks targeted working memory (digit span, word list recall), visuospatial skills (auditory clock interpretation), executive function (serial arithmetic, verbal sequencing), attention (digit ordering, auditory detection), and language processing (verbal memory and fluency), with task difficulty progressively increased every four weeks. Ten dual-task trials were performed per session.

Intensity control: Aerobic training was conducted on a treadmill at 55–70% of age-predicted maximum heart rate (HRmax), verified with Polar ^®^ chest-strap monitors and a Borg Rating of Perceived Exertion (RPE) of 11–13. Resistance exercises were performed at 50–69% of one-repetition maximum (1-RM) using color-coded resistance bands for major upper- and lower-limb joints. 1-RM testing was repeated biweekly, and heart rate and RPE were monitored every 10 minutes to ensure adherence to prescribed intensity.

### Control group

Participants received standard conventional physiotherapy of equal frequency, duration, and intensity but without cognitive or blindfold components. Aerobic training was performed on a treadmill at 55–70% HRmax, continuously monitored with heart-rate telemetry and maintained at an RPE of 11–13. Resistance exercises targeted the same muscle groups at 50–69% 1-RM, with biweekly 1-RM reassessments and progressive load adjustments using resistance bands. Heart rate and RPE were monitored every 10 minutes to maintain the target intensity.

### Outcome measures

The primary outcome was cognitive function measured by the Montreal Cognitive Assessment (MoCA) at baseline of 0 ^th^ week and post-intervention at 12 ^th^ week. Cognitive function was assessed using the Montreal Cognitive Assessment (MoCA), a widely used 30-point screening tool designed to detect mild cognitive impairment across multiple cognitive domains. The MoCA evaluates the following subdomains: executive functions (e.g., alternating trail making, verbal abstraction, clock drawing), memory (immediate and delayed recall of a word list), language (naming, sentence repetition, verbal fluency), attention (digit span, vigilance, serial subtraction), visuospatial abilities (cube and clock copying), abstraction, and orientation to time and place. Administration typically takes 10–15 minutes. Scores below 26 indicate potential cognitive impairment. The MoCA is more sensitive than the Mini-Mental State Examination (MMSE) for detecting subtle cognitive deficits, making it suitable for assessing cognitive function in clinical populations, including individuals with type 2 diabetes mellitus. ^41^

### Interim analysis and safety monitoring

Participant adherence, safety, and adverse events were monitored continuously, with interim analyses conducted monthly to evaluate compliance, efficacy signals, and participant well-being.

### Statistical analysis

Statistical analysis was performed using IBM SPSS Statistics 30 version ^19^ under subscription version. with a two-tailed alpha level of 0.05 defining significance. Normality of data distribution was confirmed via Shapiro-Wilk tests ( *W* > 0.90 for all groups). Within-group changes in MoCA scores were analyzed using paired *t*-tests, while between-group differences at post-intervention were assessed via independent *t*-tests. Effect sizes were calculated using Cohen’s *d*, interpreted as small ( *d* = 0.20), medium ( *d* = 0.50), and large ( *d* ≥ 0.80). ^42^ Homogeneity of variance was verified with Levene’s test ( *p* > 0.10 for all comparisons), supporting the use of equal variances assumed in *t*-tests. Clinical significance was evaluated against the established minimal clinically important difference (MCID) of 2.3 points for MoCA in diabetic populations. All data are reported as mean ± standard deviation (SD), with 95% confidence intervals (CI) calculated for mean differences.

## Results

A total of 62 participants diagnosed with type 2 diabetes mellitus were randomly allocated into two groups: Group A (Cognitive-Motor Dual-Task Blindfold Training, CMDBT; n = 31) and Group B (Control: Moderate-Intensity Aerobic and Resistance Training; n = 31). Baseline demographic and clinical characteristics were comparable between groups, with no statistically significant differences observed in age, gender distribution, duration of diabetes, educational level, HbA1c, body mass index (BMI), or Mini-Mental State Examination (MMSE) scores (all p > 0.05), confirming homogeneity. ^43^ The normality of Montreal Cognitive Assessment (MoCA) scores was verified using the Shapiro-Wilk test (p > 0.05), allowing the use of parametric statistical analyses. There were no dropouts during the intervention period, and all participants completed baseline and post-intervention assessments. Data were analyzed on an intention-to-treat basis.

**Figure 2.**
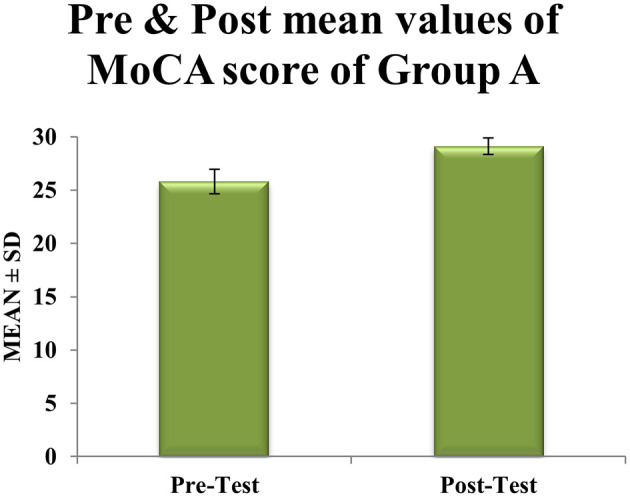
Graphical representation of Means of pre and post values of MoCA within experimental Group A.

**Figure 3.**
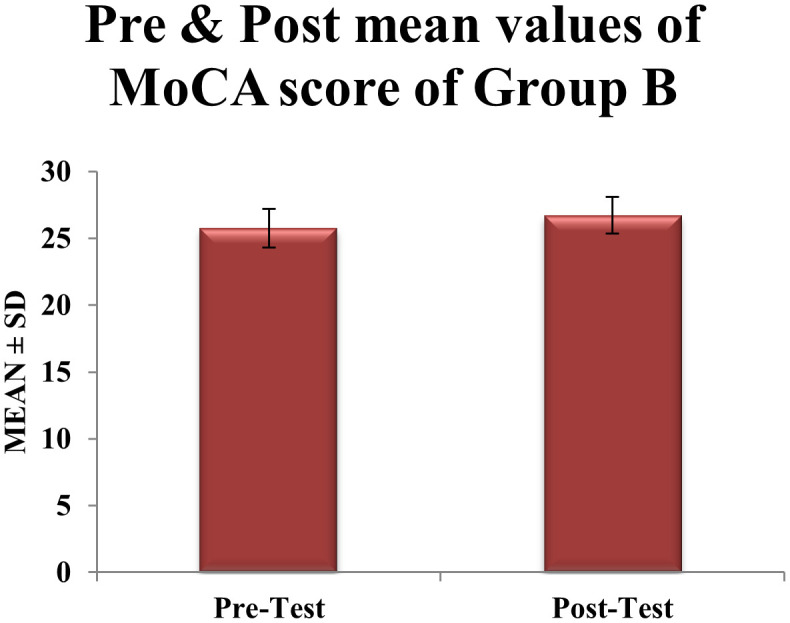
Graphical representation of Means of pre and post values of MoCA within CONTROL Group B.

Within-group analyses showed significant improvements in cognitive function for both groups. In Group A (CMDBT), mean MoCA scores increased by 3.32 points, from 25.81 ± 1.74 pre-intervention to 29.13 ± 0.76 post-intervention (t = 6.32, df = 30, p < 0.0001). This increase exceeded the minimal clinically important difference (MCID) of 2.3 points and was accompanied by a 56% reduction in score variability (SD reduced from 1.74 to 0.76), indicating a consistent and robust treatment effect ( Table 1, Figure 2). In contrast, **Group B (control)** exhibited a statistically significant but smaller mean increase of **0.94 points** in MoCA scores, from 25.77 ± 1.45 to 26.71 ± 1.37 (t = 6.02, df = 30, p = 0.0006). However, this gain did not surpass the MCID threshold. The standard deviation decreased marginally by about 5.5% (from 1.45 to 1.37), suggesting more variability in response to aerobic and resistance training alone ( Table 2, Figure 3).

**Table 1.** Pre & post Mean score values of MoCA scale within experimental group A.

Test	N	Mean score	Standard deviation	DF	t-value	p-value	Std. Error
Pre	31	25.81	1.14	30	15.87	0.0001	0.28
Post	31	29.13	0.76				

The data in Table 1 show the pre and post values of Montreal Cognitive Assessment (MoCA) scale in experimental group A.

**Table 2.** Pre & post Mean score values of MoCA scale within CONTROL group B.

Test	N	Mean score	Standard deviation	DF	t-value	p-value	Std. Error
Pre	31	25.77	1.45	30	6.10	0.0001	0.15
Post	31	26.71	1.37				

The data in Table 2 show the pre and post test mean values of Montreal Cognitive Assessment (MoCA) scale in CONTROL group B.

**Figure 4.**
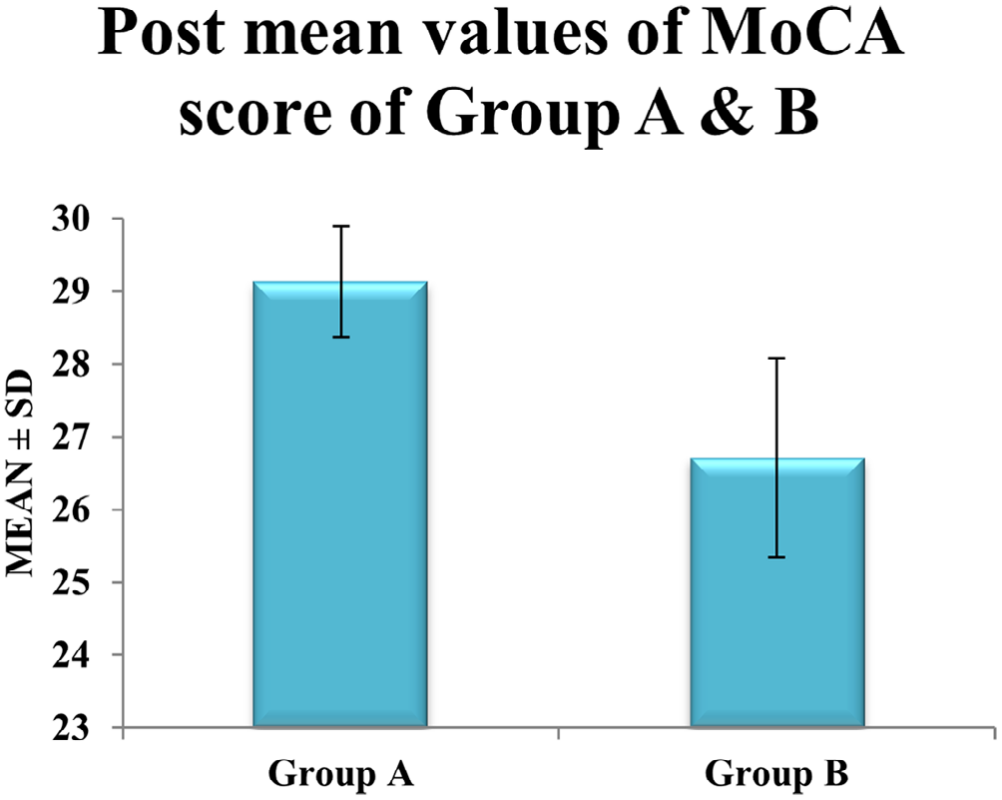
Graphical representation of Means of post values of MoCA between Group A & Group B.

Between-group comparison demonstrated that **post-intervention MoCA scores in Group A (29.13 ± 0.76) were significantly higher than in Group B (26.71 ± 1.37)**, with a mean difference of **2.42 points** (p < 0.0001) (see Table 3). Effect size analysis showed a large effect for Group A (Cohen’s d = 1.89) versus a moderate effect for Group B (d = 0.65) ( Figure 3), further supporting the superior cognitive benefits of the blindfolded cognitive-motor dual-task training ( Table 3, Figure 4).

**Table 3.** Mean score of post-interventional values of MONTREAL COGNITIVE ASSESSMENT (MoCA) between experimental groups A, and control group B.

Test	N	Mean score	Standard deviation	DF	t-value	p-value	Std. Error
Post	31	29.13	0.76	30	8.58	0.0001	0.28
Post	31	26.71	1.37				

The data in Table 3 show the post-mean values of the Montreal Cognitive Assessment Scale for Groups A and B.

## Discussion

The primary research question of this study was whether incorporating visual deprivation into cognitive-motor dual-task training (CMDBT) would produce superior cognitive improvements in adults with type 2 diabetes mellitus (T2DM) compared to standard conventional therapy comprising moderate-intensity aerobic and resistance exercises. The present study demonstrated that CMDBT significantly improved overall cognitive function, with participants in the experimental group showing greater gains than those undergoing conventional therapy alone. Both interventions yielded statistically significant pre-to-post improvements (p < 0.0001), but the magnitude of change in the CMDBT group exceeded the minimal clinically important difference (MCID) for the Montreal Cognitive Assessment (MoCA), highlighting the additive benefits of integrating cognitive-motor tasks with visual deprivation.

Cognitive decline in T2DM is multifactorial, with vascular dysfunction and metabolic dysregulation playing critical roles. Chronic hyperglycemia induces glycation of vascular proteins, endothelial dysfunction, and oxidative stress, which impair cerebral perfusion and neurovascular coupling, ultimately leading to neuronal injury and cognitive deterioration. ^44, 45^ These pathophysiological processes predominantly affect executive function, memory, processing speed, verbal fluency, and global cognition, consistent with deficits observed in diabetic populations. ^46, 47^ By situating the study’s findings within this mechanistic framework, the superior cognitive gains observed in the CMDBT group can be understood as counteracting diabetes-related neurovascular and metabolic insults through targeted neuroplastic stimulation.

The cognitive improvements in the CMDBT group are likely mediated by enhanced functional connectivity between motor and cognitive brain networks elicited by dual-task training. Walking on a treadmill while performing cognitive tasks engages multiple neural circuits—including the prefrontal cortex, basal ganglia, cerebellum, and brainstem—promoting neuroplasticity through task-specific integration of sensory, motor, and cognitive inputs. ^48, 49^ The addition of blindfolding enforces sensory substitution, rerouting non-visual information via tactile and proprioceptive pathways to the visual cortex, thereby enhancing cross-modal plasticity and interhemispheric communication. ^50, 51^ This mechanism aligns with evidence from sensory substitution research demonstrating cortical reorganization and improved functional outcomes in visually impaired and neurologically rehabilitated individuals. ^52, 53^

Previous studies support the efficacy of multimodal cognitive-motor interventions in older adults. Eggenberger et al. ^48^ investigated combined cognitive and physical training in adults ≥70 years and reported significant improvements in working memory, executive function, and attention, particularly with longer-duration interventions. Similarly, Hewston and Deshpande ^49^ found that dual-task balance training improved gait performance and reduced cognitive-motor interference in adults with T2DM, indirectly supporting neurological benefits from dual-task approaches. Our findings extend this literature by demonstrating that adding visual deprivation to cognitive-motor training further amplifies cognitive gains, likely through enhanced multisensory integration and compensatory neural activation.

This study advances current knowledge by incorporating blindfold-induced sensory deprivation into structured CMDBT sessions, combined with moderate-intensity aerobic and resistance training. The intervention was feasible, time-efficient, and cost-effective, making it suitable for clinical and rehabilitation settings. Clinically, the magnitude of cognitive improvement in the CMDBT group aligns with thresholds associated with reduced dementia risk in diabetic populations, emphasizing the potential utility of sensory-enhanced cognitive-motor interventions to mitigate diabetes-associated cognitive decline.

In conclusion, integrating CMDBT with visual deprivation appears to offer significant advantages over conventional therapy for improving cognitive outcomes in adults with T2DM. These results advocate for incorporating sensory-enhanced dual-task training into rehabilitation protocols. Future research should investigate long-term cognitive and functional outcomes, optimal dosing, and underlying neurophysiological mechanisms using neuroimaging and biomarker analyses.

## Conclusion

This study demonstrated that a 12-week cognitive-motor dual-task training (CMDBT) program, combined with aerobic and resistance exercises, produced significant and clinically meaningful improvements in cognitive function among individuals with type 2 diabetes mellitus (T2DM). While both CMDBT and moderate-intensity aerobic exercise interventions yielded significant within-group cognitive gains, CMDBT resulted in significantly greater enhancements compared to aerobic training alone. These findings highlight CMDBT’s potential as an effective, feasible, and time-efficient intervention to mitigate cognitive decline associated with T2DM. The robust improvements observed provide a strong rationale for integrating CMDBT into clinical rehabilitation protocols and warrant further research to explore its long-term benefits and underlying neurophysiological mechanisms. Ultimately, such interventions may contribute to improving cognitive health and overall quality of life in patients living with T2DM.

### Limitations of the study

• The study includes small sample size, the study did not include long term follow up.
• This study sample size was relatively small to detect the effects of cognitive motor dual-task training (CMDTT) on cognitive function in patients with type 2 diabetes mellitus.

### Recommendations of the study

• Follow-up programs can be included to assess the short- and long-term effects of the treatment.
• Further studies should be conducted to evaluate the effects of cognitive motor dual-task training in other conditions.
• The effects of cognitive motor dual-task training on other types of diabetes and its complications should be studied.
• Further study should include more measurement tools like fMRI.

## Ethics and consent statement

This study was conducted in accordance with the Declaration of Helsinki and was approved by an institutional ethics committee on 30-12-2023 at the Apollo Institute of Medical Science and Research, Chittoor, Andhra Pradesh, India. Ethics Committee Number: **PG/35/IEC/AIMSR/2023.** Written informed consent was obtained from all participants. The study conducted as per guideline of Declaration of Helsinki.

**Table:** DATA COLLECTION SHEET: the study participants are De identified with the serial number

EXPERIMENTAL GROUP A - CMDBT			CONTROL GROUP B - Conventional Therapy		
S.no	MoCA Score		S.no	MoCA Score	
	Pre-Test	Post-Test		Pre-Test	Post-Test
1	24	28	1	25	26
2	25	29	2	27	28
3	24	29	3	25	26
4	27	30	4	24	25
5	27	30	5	27	25
6	23	28	6	25	27
7	24	29	7	24	24
8	24	28	8	26	27
9	26	30	9	27	28
10	24	29	10	24	25
11	27	29	11	28	29
12	29	30	12	26	28
13	26	29	13	28	28
14	28	30	14	26	27
15	27	30	15	24	27
16	24	28	16	26	27
17	25	29	17	27	28
18	24	28	18	24	26
19	28	30	19	26	26
20	26	29	20	28	28
21	28	30	21	24	25
22	28	30	22	27	28
23	25	29	23	26	26
24	23	28	24	26	27
25	27	30	25	28	29
26	25	29	26	24	25
27	26	29	27	26	27
28	24	28	28	24	26
29	26	29	29	24	25
30	28	29	30	28	29
31	28	30	31	25	26

## Data Availability

The datasets generated analyzed during the current study are available in the Anandh Raj, J (2025). Pretest and post test values of MoCA in Group A and B in Type 2 Diabetes Mellitus subjects. figshare. Dataset. ( https://figshare.com/s/014afef5a58e663a3b96). ^54^ **DOI:** 10.6084/m9.figshare.28513433.V2 The extended data for this study include the demographic dataset of participants have been deposited in the Anandh Raj, J (2025). Baseline characteristics of 12-week & 18th-week follow-up of cognitive motor dual-task training in type 2 diabetes mellitus subjects. figshare. Dataset. https://doi.org/10.6084/m9.figshare.29134604.v1. ^43^ Data are available under the terms of the Creative Commons Attribution 4.0 International license (CC-BY 4.0)
