# Supplementary material for: The Effect Of Visual Deprivation During Cognitive Motor Dual Task Training On Cognitive Function In Type 2 Diabetes Mellitus
Source: F1000Res. 2025 Oct 17;14:592. Originally published 2025 Jun 17. [Version 3] doi: 10.12688/f1000research.162466.3 (PMC12583909; doi:10.12688/f1000research.162466.3)
Supplement: Peer review history

# Reviewer report: Reviewer response for version 3

**DOI:** 10.5256/f1000research.189242.r425726

**Published:** 2025-11-17

**Authors:** João Gabriel da Silveira Rodrigues

**Affiliations:** 1 Universidade Federal de Minas Gerais, Belo Horizonte, State of Minas Gerais, Brazil

The manuscript entitled “The effect of visual deprivation during cognitive motor dual-task training on cognitive function in type 2 diabetes mellitus” evaluated the effectiveness of cognitive motor dual-task training (CMDBT) combined with aerobic and resistance exercises on cognitive function in individuals with type 2 diabetes mellitus (T2DM). The topic is relevant and adds valuable information regarding a novel intervention. However, although the manuscript addresses an interesting subject for the scientific community, several major issues need to be addressed before the study can be considered for publication.

 Major Comments

1. The MoCA was originally conceptualized and validated as a cognitive screening tool, not as an instrument to assess the effectiveness or efficacy of an intervention. Please justify why this screening tool was selected for evaluating intervention outcomes.
2. A previous study (DOI: 10.1016/j.diabres.2021.108700) investigated the effects of exercise training on specific cognitive domains in individuals with T2DM. The authors reported that exercise efficacy appears to be domain-specific, with improvements in attentional and executive processes, whereas processing speed and memory remained unchanged. In light of this evidence, the present manuscript should engage in a discussion with the aforementioned study to address the limitations of using the MoCA—which is a screening instrument and not designed to evaluate specific cognitive domains or intervention effects.

Methods

 3. From a physiological perspective, the repetition of acute exercise sessions can lead to chronic adaptations. Therefore, reporting adherence (i.e., the percentage of total sessions completed) for the intervention group is mandatory.

 4. Intervention studies should account for potential dropouts when determining sample size to ensure sufficient statistical power. Please clarify why this adjustment was not performed in the present study.

 5. In Figure 1, the authors should indicate how many participants were excluded during the initial recruitment phase due to the exclusion criteria.

 6. Participants in the control group received standard conventional physiotherapy. However, previous studies have highlighted that physiotherapy interventions may also enhance neurotrophic factors (DOI: 10.1016/j.archger.2011.05.014). Please clarify how this potential confounding factor was addressed or controlled for in the current study.

 7. The authors did not report the participants’ characteristics. Sociodemographic, metabolic, and cognitive characteristics of the participants were not reported. These data are essential for interpreting the findings and determining their generalizability. Please include this information.

 8. Age and educational level are well known to influence MoCA performance. Therefore, it is crucial to include these parameters, along with other relevant variables, in the proposed Table 1 describing participant characteristics.

 9. Some medications used to control blood glucose may affect cognitive function and MoCA scores. Considering the longitudinal design of this study, please include a description of the drug therapy prescribed to participants (pre- and post-intervention) to better contextualize this potential limitation.

 10. The limitation session of this study is too small. IMO, several points raised in this letter should be added as study limitations.

 11. Details of the exercise session, specially regarding the resistance exercise (exercise volume, rest, exercise number, sequence, etc) should be provided.

Is the work clearly and accurately presented and does it cite the current literature?

Partly

If applicable, is the statistical analysis and its interpretation appropriate?

Yes

Are all the source data underlying the results available to ensure full reproducibility?

No

Is the study design appropriate and is the work technically sound?

Yes

Are the conclusions drawn adequately supported by the results?

Yes

Are sufficient details of methods and analysis provided to allow replication by others?

No

Reviewer Expertise:

Physical exercise, exercise physiology, cognition, type 2 diabetes, older adults

I confirm that I have read this submission and believe that I have an appropriate level of expertise to state that I do not consider it to be of an acceptable scientific standard, for reasons outlined above.

# Reviewer report: Reviewer response for version 3

**DOI:** 10.5256/f1000research.189242.r425730

**Published:** 2025-11-07

**Authors:** Maria Rizzo

**Affiliations:** 1 Messina University, Messina, Italy

The manuscript "The Effect Of Visual Deprivation During Cognitive Motor Dual Task Training On Cognitive Function In Type 2 Diabetes Mellitus" presents a methodologically rigorous and innovative RCT exploring the effects of visual deprivation during cognitive-motor training in individuals with Type 2 diabetes mellitus.

 It contributes novel insights into the role of sensory deprivation in enhancing cognitive outcomes through cross-modal plasticity mechanisms.

 The study is scientifically sound, well-documented, and clinically relevant, with appropriate methodology and transparent data sharing. Only minor editorial and structural improvements are recommended for clarity. 

 1. Minor editorial improvements could further enhance readability, particularly by simplifying long sentences in the Introduction and Discussion.

 2. Although technically sound, the manuscript could benefit from a brief justification of why the 12-week intervention period was chosen, referencing similar cognitive-motor training durations from prior literature.

 3. It would be helpful to include a short paragraph in *Data Availability* summarizing how to access the dataset from *figshare* to guide readers unfamiliar with the platform.

 4. Statistical analyses are appropriate and correctly interpreted. Use of both *p*-values and effect sizes (Cohen’s *d*) is commendable. The authors verified assumptions of normality and variance homogeneity before applying parametric tests. Results are reported with adequate precision, including mean ± SD and confidence intervals. The discussion accurately reflects the magnitude and clinical relevance of findings (MCID of 2.3 points for MoCA). The Authors can add in disccussion section "Future studies could incorporate multivariate analyses (e.g., ANCOVA) to control for potential covariates such as age, baseline cognitive status, or education level."

 5. To strengthen the impact, the authors could include a short paragraph discussing potential neurophysiological mechanisms (e.g., BDNF modulation) to bridge behavioral outcomes and neural plasticity.

 6. Ensure consistency in abbreviation usage (e.g., CMDBT, CMDT).

Is the work clearly and accurately presented and does it cite the current literature?

Yes

If applicable, is the statistical analysis and its interpretation appropriate?

Yes

Are all the source data underlying the results available to ensure full reproducibility?

Yes

Is the study design appropriate and is the work technically sound?

Yes

Are the conclusions drawn adequately supported by the results?

Yes

Are sufficient details of methods and analysis provided to allow replication by others?

Yes

Reviewer Expertise:

Physiology and Behavior

I confirm that I have read this submission and believe that I have an appropriate level of expertise to confirm that it is of an acceptable scientific standard, however I have significant reservations, as outlined above.

# Reviewer report: Reviewer response for version 3

**DOI:** 10.5256/f1000research.189242.r425729

**Published:** 2025-11-03

**Authors:** Arunachalam Ramachandran,

**Affiliations:** 1 Madhav University, Abu Road, Rajasthan, India

The research is well drafted and the research question dealt with is of good clinical significance, given the fact that T2DM is on the high and that too India is becoming the capital of Diabetes. I would suggest that the author include the content on how the cognitive factors affect the socio-psychological components of the patients in the introduction to emphasise the need for the study. also, I would suggest the author incorporate the impact of "Visual Deprivation" in the treatment process.

Is the work clearly and accurately presented and does it cite the current literature?

Yes

If applicable, is the statistical analysis and its interpretation appropriate?

Yes

Are all the source data underlying the results available to ensure full reproducibility?

No source data required

Is the study design appropriate and is the work technically sound?

Yes

Are the conclusions drawn adequately supported by the results?

Yes

Are sufficient details of methods and analysis provided to allow replication by others?

Yes

Reviewer Expertise:

Neuro rehabilitation, Physiotherapy, Yoga and Virtual rehabilitation

I confirm that I have read this submission and believe that I have an appropriate level of expertise to confirm that it is of an acceptable scientific standard.

# Reviewer report: Reviewer response for version 3

**DOI:** 10.5256/f1000research.189242.r425022

**Published:** 2025-10-27

**Authors:** Dr Kiruthika Selvakumar

**Affiliations:** 1 Universiti Tunku Abdul Rahman, Sungai Long, Selangor, Malaysia

No further remarks.Approved

Is the work clearly and accurately presented and does it cite the current literature?

Partly

If applicable, is the statistical analysis and its interpretation appropriate?

Partly

Are all the source data underlying the results available to ensure full reproducibility?

Yes

Is the study design appropriate and is the work technically sound?

Partly

Are the conclusions drawn adequately supported by the results?

Partly

Are sufficient details of methods and analysis provided to allow replication by others?

Partly

Reviewer Expertise:

Physiotherapy, Headache, Migraine, Stroke Rehabilitation, Paediatric Rehabilitation, Geriatric Rehabilitation, Scoliosis and Low pain pain

I confirm that I have read this submission and believe that I have an appropriate level of expertise to confirm that it is of an acceptable scientific standard.

# Reviewer report: Reviewer response for version 2

**DOI:** 10.5256/f1000research.184769.r407289

**Published:** 2025-09-16

**Authors:** Nilton João Chantre Leite

**Affiliations:** 1 University of Évora, Évora, Portugal

Thank you for the opportunity to review this interesting manuscript. I have some recommendations to strengthen the presentation and impact of this work.

 ABSTRACT:

 1. Background: The connection you make between dual-task training and cognitive function is very persuasive. To further focus the reader, please consider adding the objective of the study.

 2. Methods: The methodology description is clear, but it might benefit from a slight reorganization to improve flow. It could be more straightforward to begin with the study design and randomization, then describe the interventions assigned to each group, and finally the assessment time points.

 3. Results: Can the main message be condensed into a single sentence, integrating the " *p"* value and the average difference observed between the groups?

 INTRODUCTION:

 1. I recommend reviewing the use of abbreviations throughout the manuscript. For clarity and consistency, ensure that each term is written in full upon its first appearance and then used consistently in all subsequent sections.

 2. In the first paragraph, I recommend updating the epidemiological data on diabetes (e.g., prevalence, mortality, economic impact) using the latest figures from the International Diabetes Federation's *Diabetes Atlas*."

  3. Please add a supporting citation for the claim that  “… *blindfold training may uniquely amplify compensatory brain activation and multisensory integration in this population. By forcing dependence on non-visual sensory modalities, blindfold cognitive-motor dual-task training may serve as a stronger neuroplastic stimulus than training allowing visual input.*

 METHODS:

 1. Please consider relocating the first paragraph to the Introduction section and the paragraph on ethical approval to the Study Design section.

 2. The sample size calculation is clearly presented. To further strengthen this section, could you please add a citation for the software/statistical method used or reference of the study from which the parameters were derived?

 3. Please  consider describing how the intensity of the interventions was controlled, and monitored across all participants and training sessions.

 4. Please clarify whether participants' baseline physical activity levels were assessed and if activity was and  controlled for during the trial. The interpretation of the results would be significantly strengthened by ruling out physical activity as a confounding variable. If this data is available, please present it. If not, this should be acknowledged as a study limitation, as differences in activity levels could offer an alternative explanation for the differential cognitive outcomes observed between the groups.

 5. Please provide a description of the MoCA instrument and its specific subdomains (e.g., executive functions, memory, language, orientation, visuospatial abilities) that were assessed. This is essential for the reader to understand what cognitive functions are being analyzed.

 STATISTICAL ANALYSIS:

 1. Please consider adding a citation for the Cohen's effect size cut-off values used.

 RESULTS:

 1. I could not locate the baseline demographic and clinical characteristics in the cited source, [Anandh Raj J: Pretest and post test values of MoCA in Group A and B in Type 2 Diabetes Mellitus subjects. Dataset. figshare. 2025]. Please review this citation for accuracy.

 2. A stated aim of this study, introduced in paragraph 7 of the Introduction, was to explore effects on MoCA subdomains. To fully address this aim, please present the analysis of these subdomains (e.g., executive functions, memory, language, orientation) in the Results section. This data is essential to provide a nuanced interpretation of the intervention's specific cognitive effects.

 3. Please consider relocating the following paragraphs to the Discussion Section:

*"Clinically, the magnitude of improvement in Group A aligns with thresholds associated with reduced dementia risk in diabetic populations, highlighting the potential of integrating sensory deprivation to augment cognitive rehabilitation in type 2 diabetes mellitus."*

*"These findings advocate for incorporating sensory-enhanced cognitive-motor dual-task training into rehabilitation protocols to optimize cognitive outcomes and potentially mitigate diabetes-associated cognitive decline."*

 DISCUSSION:

 1. To improve the focus of the Discussion, I recommend opening the section with the study's main research question. This will provide essential context for the interpretation of the findings that follow.

 2. The arguments presented in the second and third paragraphs require supporting references. Please integrate citations from the relevant literature.

Is the work clearly and accurately presented and does it cite the current literature?

Partly

If applicable, is the statistical analysis and its interpretation appropriate?

Partly

Are all the source data underlying the results available to ensure full reproducibility?

Partly

Is the study design appropriate and is the work technically sound?

Partly

Are the conclusions drawn adequately supported by the results?

Partly

Are sufficient details of methods and analysis provided to allow replication by others?

Partly

Reviewer Expertise:

Sport Medicine, Geriatrics, Sport Psychology

I confirm that I have read this submission and believe that I have an appropriate level of expertise to confirm that it is of an acceptable scientific standard, however I have significant reservations, as outlined above.

# Reviewer report: Reviewer response for version 2

**DOI:** 10.5256/f1000research.184769.r406650

**Published:** 2025-08-26

**Authors:** Dr Kiruthika Selvakumar

**Affiliations:** 1 Universiti Tunku Abdul Rahman, Sungai Long, Selangor, Malaysia

The authors have addressed all the suggested revisions. No further remarks.

Is the work clearly and accurately presented and does it cite the current literature?

Partly

If applicable, is the statistical analysis and its interpretation appropriate?

Partly

Are all the source data underlying the results available to ensure full reproducibility?

Yes

Is the study design appropriate and is the work technically sound?

Partly

Are the conclusions drawn adequately supported by the results?

Partly

Are sufficient details of methods and analysis provided to allow replication by others?

Partly

Reviewer Expertise:

Physiotherapy, Headache, Migraine, Stroke Rehabilitation, Paediatric Rehabilitation, Geriatric Rehabilitation, Scoliosis and Low pain pain

I confirm that I have read this submission and believe that I have an appropriate level of expertise to confirm that it is of an acceptable scientific standard.

# Reviewer report: Reviewer response for version 1

**DOI:** 10.5256/f1000research.178674.r395444

**Published:** 2025-07-28

**Authors:** Dr Kiruthika Selvakumar

**Affiliations:** 1 Universiti Tunku Abdul Rahman, Sungai Long, Selangor, Malaysia

Dear Editor-in-Chief/ Authors,

 Enclosed is the suggestions for further consideration. 

**Introduction:**

 The introduction is comprehensive and well-structured, covering the relationship between variables; however, the authors can consider the following suggestions:

1. The aim of the study/research gap is weakly stated, and the hypothesis is only brief.
2. The background lacks a research question and objective.
3. Rationale for using blindfold training specifically for the T2DM population is not clear.
4. Definition and clarification on CMBT are introduced towards the end; the physiology behind this training and how it is different from dual-task or physical cognitive training approaches would strengthen the objective of the study.

**Methods:**

1. The grammatical errors in the methodology are to be corrected.
2. The sample size included was 62, but the sample size calculation methods or dropout calculations were not provided in this study.
3. Since the study design is RCT, whether the study followed the CONSORT guidelines is not clear.
4. The study lacks an explanation of blinding, primary, and secondary outcome measures.
5. Details on allocation concealment mechanism, randomisation allocation, generation or enrolment were discussed.
6. Since both groups underwent training, an interim analysis including safety, efficacy and compliance checks can be included.

**Results:**

1. Inconsistency in terminologies described (the methodology mentions group A, but the results section mentions it as group 1).
2. Poor grammar and sentence construction.
3. The normality of data is not included.
4. Did the study encounter any dropouts? If so, how was the data managed for result analysis?
5. Data collection sheet – the title of the table – error to be corrected.

**Discussion:**

1. The paragraph structure is inconsistent or intermixed with literature and not clearly separated.
2. The statement “T2DM patients’ cognitive deterioration is largely due to vascular damage caused by protein accumulation on the blood vessel walls” is a pathological explanation lacking citation and not a study result, but it’s included in the results section.
3. Statement “This demonstrates CMDBT’s potential as a successful cognitive rehabilitation intervention for patients with type 2 diabetes” is vague and needs rephrasing.
4. Authors are highly encouraged to look at the sentence restructuring and phrasing.
5. Weak critical comparison with literature: especially Eggenberger et al. (2015) and Hewston et al. (2013), whether these studies align or diverge with the current study, and justification for this is not included.
6. Some of the references need to be updated (example: 2004, 2005, 2008).

Is the work clearly and accurately presented and does it cite the current literature?

Partly

If applicable, is the statistical analysis and its interpretation appropriate?

Partly

Are all the source data underlying the results available to ensure full reproducibility?

Yes

Is the study design appropriate and is the work technically sound?

Partly

Are the conclusions drawn adequately supported by the results?

Partly

Are sufficient details of methods and analysis provided to allow replication by others?

Partly

Reviewer Expertise:

Physiotherapy, Headache, Migraine, Stroke Rehabilitation, Paediatric Rehabilitation, Geriatric Rehabilitation, Scoliosis and Low pain pain

I confirm that I have read this submission and believe that I have an appropriate level of expertise to confirm that it is of an acceptable scientific standard, however I have significant reservations, as outlined above.

# Response

**Published:** 2025-08-01

**Authors:** J Anandh Raj

Dear Editor and Reviewer,

 We thank you for your constructive review and helpful suggestions. We have addressed each point in detail below and revised the manuscript accordingly to improve clarity, rigor, and scientific quality.

**Introduction**

- We have clarified the research aim, hypothesis, and rationale earlier in the introduction as requested. The rationale for blindfold training in T2DM is now more explicitly explained with supporting citations. CMDBT is defined and distinguished from other dual-task approaches earlier in the text.

**Methods**

- Grammatical errors have been corrected.
- A detailed sample size calculation with dropout considerations is added.
- CONSORT adherence is clearly stated, and a flow diagram is included (Figure 1).
- Blinding procedures and primary outcome measures are described more explicitly.
- Randomization and allocation concealment methods are clarified.
- Interim safety, compliance monitoring details are included.

**Results**

- Group terminology is now consistent throughout (Group A and B).
- Grammar and sentence structure are improved for clarity.
- Normality testing results (Shapiro-Wilk) are added.
- No dropouts occurred; analysis was intention-to-treat; this is now clearly stated.
- Table titles and figure captions have been corrected.

**Discussion**

- Paragraphs are restructured for better flow and separation of study findings, literature context, and mechanisms.
- Pathophysiological explanations are moved from results to discussion.
- Vague statements are rephrased for precision.
- Literature comparisons with Eggenberger et al. and Hewston et al. include discussion on alignment and divergence.
- Some outdated references have been updated with recent literature.

We appreciate the opportunity to improve our manuscript based on your expert feedback and believe the revisions have enhanced the quality and transparency of the work.

 Sincerely,

 J Anandh Raj

 On behalf of all authors

# Response

**Published:** 2025-10-02

**Authors:** J Anandh Raj

Dear Reviewers,

 We sincerely thank you for the thorough and constructive feedback on our manuscript. We greatly appreciate the time and effort you have taken to provide valuable suggestions. We have carefully revised the manuscript in line with your comments, and below we provide a detailed, point-by-point response.

**Introduction**

1. **Aim of the study and hypothesis**:
  Thank you for this observation. We have revised the introduction to clearly state the research gap, aim, and a more explicit hypothesis.
2. **Research question and objective**:
  We have added a clearly defined research question and an explicit objective at the end of the introduction section.
3. **Rationale for blindfold training in T2DM population**:
  Additional explanation has been incorporated to clarify why blindfold training may enhance sensory substitution and cognitive engagement specifically in T2DM patients, supported by relevant references.
4. **Clarification of CMDBT definition and physiology**:
  We have restructured the introduction to define CMDBT earlier and included an explanation of its underlying physiological basis, highlighting how it differs from conventional dual-task or physical-cognitive training.

**Methods**

1. **Grammatical corrections**:
  The methodology section has been thoroughly revised for grammar and clarity.
2. **Sample size calculation**:
  We have now provided details of the sample size calculation method, including assumptions used, and clarified dropout considerations.
3. **CONSORT guidelines**:
  We have revised the manuscript to state that the trial adhered to CONSORT guidelines, and included a flow diagram in the supplementary material.
4. **Blinding and outcomes**:
  The revised manuscript specifies the type of blinding employed (assessor blinding), and explicitly lists the primary and secondary outcome measures.
5. **Randomisation and allocation concealment**:
  Details on random sequence generation, allocation concealment, and enrolment procedures have been added for clarity.
6. **Interim analysis**:
  A statement has been added explaining compliance and safety monitoring during the intervention, though no formal interim efficacy analysis was planned.

**Results**

1. **Inconsistent terminologies**:
  All group identifiers have been standardized (Group A/Group B) throughout the manuscript for consistency.
2. **Grammar and construction**:
  The results section has been carefully revised for clarity and grammar.
3. **Normality testing**:
  Details of the normality test (Shapiro–Wilk) have been added to the statistical analysis section.
4. **Dropouts**:
  Information on participant retention and dropouts, along with how missing data was handled (intention-to-treat analysis), has been included.
5. **Data collection sheet/table title error**:
  The table titles have been corrected.

**Discussion**

1. **Paragraph structure**:
  The discussion has been restructured to separate study findings from supporting literature more clearly.
2. **Pathological explanation in results section**:
  The statement regarding vascular damage was relocated to the discussion and now includes appropriate citations.
3. **Vague conclusion statement**:
  The statement on CMDBT effectiveness has been rephrased to be more precise and evidence-based.
4. **Sentence restructuring**:
  The discussion has been revised for sentence flow and clarity.
5. **Critical comparison with literature**:
  We have added a detailed comparison with Eggenberger et al. (2015) and Hewston et al. (2013), highlighting alignment and differences with our study findings.
6. **Updated references**:
  Several outdated references have been replaced with recent publications to strengthen the discussion.

**Conclusion:**

 We believe these revisions have improved the clarity, scientific rigor, and overall quality of the manuscript. We thank the reviewers once again for their valuable input, which has substantially strengthened our work.

 Respectfully,

 J Anandh Raj
